# Cerebral microbleeds and stroke risk after ischaemic stroke or transient ischaemic attack: a pooled analysis of individual patient data from cohort studies

**DOI:** 10.1016/S1474-4422(19)30197-8

**Published:** 2019-07

**Authors:** Duncan Wilson, Gareth Ambler, Keon-Joo Lee, Jae-Sung Lim, Masayuki Shiozawa, Masatoshi Koga, Linxin Li, Caroline Lovelock, Hugues Chabriat, Michael Hennerici, Yuen Kwun Wong, Henry Ka Fung Mak, Luis Prats-Sánchez, Alejandro Martínez-Domeño, Shigeru Inamura, Kazuhisa Yoshifuji, Ethem Murat Arsava, Solveig Horstmann, Jan Purrucker, Bonnie Yin Ka Lam, Adrian Wong, Young Dae Kim, Tae-Jin Song, Maarten Schrooten, Robin Lemmens, Sebastian Eppinger, Thomas Gattringer, Ender Uysal, Zeynep Tanriverdi, Natan M Bornstein, Einor Ben Assayag, Hen Hallevi, Jun Tanaka, Hideo Hara, Shelagh B Coutts, Lisa Hert, Alexandros Polymeris, David J Seiffge, Philippe Lyrer, Ale Algra, Jaap Kappelle, Rustam Al-Shahi Salman, Hans R Jäger, Gregory Y H Lip, Heinrich P Mattle, Leonidas D Panos, Jean-Louis Mas, Laurence Legrand, Christopher Karayiannis, Thanh Phan, Sarah Gunkel, Nicolas Christ, Jill Abrigo, Thomas Leung, Winnie Chu, Francesca Chappell, Stephen Makin, Derek Hayden, David J Williams, M Eline Kooi, Dianne H K van Dam-Nolen, Carmen Barbato, Simone Browning, Kim Wiegertjes, Anil M Tuladhar, Noortje Maaijwee, Christine Guevarra, Chathuri Yatawara, Anne-Marie Mendyk, Christine Delmaire, Sebastian Köhler, Robert van Oostenbrugge, Ying Zhou, Chao Xu, Saima Hilal, Bibek Gyanwali, Christopher Chen, Min Lou, Julie Staals, Régis Bordet, Nagaendran Kandiah, Frank-Erik de Leeuw, Robert Simister, Aad van der Lugt, Peter J Kelly, Joanna M Wardlaw, Yannie Soo, Felix Fluri, Velandai Srikanth, David Calvet, Simon Jung, Vincent I H Kwa, Stefan T Engelter, Nils Peters, Eric E Smith, Yusuke Yakushiji, Dilek Necioglu Orken, Franz Fazekas, Vincent Thijs, Ji Hoe Heo, Vincent Mok, Roland Veltkamp, Hakan Ay, Toshio Imaizumi, Beatriz Gomez-Anson, Kui Kai Lau, Eric Jouvent, Peter M Rothwell, Kazunori Toyoda, Hee-Joon Bae, Joan Marti-Fabregas, David J Werring, Microbleeds International Collaborative Network, Kirsty Harkness, Louise Shaw, Jane Sword, Azlisham Mohd Nor, Pankaj Sharma, Deborah Kelly, Frances Harrington, Marc Randall, Matthew Smith, Karim Mahawish, Abduelbaset Elmarim, Bernard Esisi, Claire Cullen, Arumug Nallasivam, Christopher Price, Adrian Barry, Christine Roffe, John Coyle, Ahamad Hassan, Jonathan Birns, David Cohen, Lakshmanan Sekaran, Adrian Parry-Jones, Anthea Parry, David Hargroves, Harald Proschel, Prabel Datta, Khaled Darawil, Aravindakshan Manoj, Mathew Burn, Chris Patterson, Elio Giallombardo, Nigel Smyth, Syed Mansoor, Ijaz Anwar, Rachel Marsh, Sissi Ispoglou, Dinesh Chadha, Mathuri Prabhakaran, Sanjeevikumar Meenakishundaram, Janice O'Connell, Jon Scott, Vinodh Krishnamurthy, Prasanna Aghoram, Michael McCormick, Nikola Sprigg, Paul O'Mahony, Martin Cooper, Lillian Choy, Peter Wilkinson, Simon Leach, Sarah Caine, Ilse Burger, Gunaratam Gunathilagan, Paul Guyler, Hedley Emsley, Michelle Davis, Dulka Manawadu, Kath Pasco, Maam Mamun, Robert Luder, Mahmud Sajid, Ijaz Anwar, James Okwera, Elizabeth Warburton, Kari Saastamoinen, Timothy England, Janet Putterill, Enrico Flossman, Michael Power, Krishna Dani, David Mangion, Appu Suman, John Corrigan, Enas Lawrence, Djamil Vahidassr, Clare Shakeshaft, Martin Brown, Andreas Charidimou, Hannah Cohen, Gargi Banerjee, Henry Houlden, Mark White, Tarek Yousry, Kirsty Harkness, Enrico Flossmann, Nigel Smyth, Louise Shaw, Elizabeth Warburton, Keith Muir, Marwan El-Koussy, Pascal Gratz, Jeremy Molad, Amos Korczyn, Efrat Kliper, Philippe Maeder, Achim Gass, Chahin Pachai, Luc Bracoub, Marie-Yvonne Douste-Blazy, Marie Dominique Fratacci, Eric Vicaut, Shoichiro Sato, Kaori Miwa, Kyohei Fujita, Toshihiro Ide, Henry Ma, John Ly, Shahoo Singhal, Ronil Chandra, Lee-Anne Slater, Cathy Soufan, Christopher Moran, Christopher Traenka, Sebastian Thilemann, Joachim Fladt, Henrik Gensicke, Leo Bonati, Beom Joon Kim, Moon-Ku Han, Jihoon Kang, Eunbin Ko, Mi Hwa Yang, Myung Suk Jang, Sean Murphy, Fiona Carty, Layan Akijian, John Thornton, Mark Schembri, Elles Douven, Raquel Delgado-Mederos;, Rebeca Marín, Pol Camps-Renom, Daniel Guisado-Alonso, Fidel Nuñez, Santiago Medrano-Martorell, Elisa Merino, Kotaro Iida, Syuhei Ikeda, Masashi Nishihara, Hiroyuki Irie, Derya Selcuk Demirelli, Jayesh Modi Medanta, Charlotte Zerna, Maria Valdés Hernández, Paul Armitage, Anna Heye, Susana Muñoz-Maniega, Eleni Sakka, Michael Thrippleton, Martin Dennis, Ysoline Beigneux, Mauro Silva, Narayanaswamy Venketasubramanian, Shu Leung Ho, Raymond Tak Fai Cheung, Koon Ho Chan, Kay Cheong Teo, Edward Hui, Joseph Shiu Kwong Kwan, Richard Chang, Man Yu Tse, Chu Peng Hoi, Chung Yan Chan, Oi Ling Chan, Ryan Hoi Kit Cheung, Edmund Ka Ming Wong, Kam Tat Leung, Suk Fung Tsang, Hing Lung Ip, Sze Ho Ma, Karen Ma, Wing Chi Fong, Siu Hung Li, Richard Li, Ping Wing Ng, Kwok Kui Wong, Wenyan Liu, Lawrence Wong, Lino Ramos, Els De Schryver, Joost Jöbsis, Jaap van der Sande, Paul Brouwers, Yvo Roos, Jan Stam, Stef Bakker, Henk Verbiest, Wouter Schoonewille, Cisca Linn, Leopold Hertzberger, Maarten van Gemert, Paul Berntsen, Jeroen Hendrikse, Paul Nederkoorn, Werner Mess, Peter Koudstaal, Alexander Leff, Nicholas Ward, Parashkev Nachev, Richard Perry, Hatice Ozkan, John Mitchell

**Affiliations:** a Stroke Research Centre, Department of Brain Repair and Rehabilitation, UCL Queen Square Institute of Neurology, London, UK; b National Hospital for Neurology and Neurosurgery, London UK; c New Zealand Brain Research Institute, Christchurch, New Zealand; d Department of Statistical Science, University College London, London, UK; e Department of Neurology, Seoul National University Bundang Hospital, Seoul National University School of Medicine, Seongnam, South Korea; f Department of Neurology, Hallym University Sacred Heart Hospital, Anyang, South Korea; g Department of Cerebrovascular Medicine, National Cerebral and Cardiovascular Center, Suita, Osaka, Japan; h Centre for Prevention of Stroke and Dementia, University of Oxford, Oxford, UK; i Assistance Publique Hôpitaux de Paris, Lariboisière Hospital, Department of Neurology, Paris, France; j Département Hospitalo-Universtaire NeuroVasc, University Paris Diderot, and INSERM U1141, Paris, France; k Department of Neurology, Universitätsmedizin Mannheim, University of Heidelberg, Mannheim, Germany; l Division of Neurology, Department of Medicine, The University of Hong Kong, Hong Kong; m Department of Diagnostic Radiology, The University of Hong Kong, Hong Kong; n Department of Neurology, Hospital de la Santa Creu i Sant Pau, Biomedical Research Institute, Barcelona, Spain; o Department of Neurosurgery, Kushiro City General Hospital, Kushiro, Japan; p Departments of Neurology and Radiology, Massachusetts General Hospital, Harvard Medical School, Boston MA, USA; q Department of Neurology, Heidelberg University Hospital, Heidelberg, Germany; r Therese Pei Fong Chow Research Centre for Prevention of Dementia, Gerald Choa Neuroscience Centre, Lui Che Woo Institute of Innovative Medicine, Department of Medicine and Therapeutics, The Chinese University of Hong Kong, Hong Kong; s Department of Neurology, Yonsei University College of Medicine, Seoul, South Korea; t Department of Neurology, Ewha Womans University College of Medicine, Seoul, South Korea; u Center for Brain and Disease Research, VIB, Leuven, Belgium; v Experimental Neurology and Leuven Institute for Neuroscience and Disease, Katholieke Universiteit Leuven, University of Leuven, Laboratory of Neurobiology, Leuven, Belgium; w Department of Neurology, Medical University of Graz, Graz, Austria; x Department of Neurology, Demiroglu Bilim University, Istanbul, Turkey; y Department of Neurology, Tel-Aviv Sourasky Medical Center, Tel-Aviv, Israel; z Sackler Faculty of Medicine, Tel-Aviv University, Tel-Aviv, Israel; aa Division of Neurology, Department of Internal Medicine, Saga University Faculty of Medicine, Nabeshima, Saga, Japan; ab Calgary Stroke Program, Department of Clinical Neurosciences, Radiology and Community Health Sciences, Hotchkiss Brain Institute, University of Calgary, Calgary, AB, Canada; ac Department of Neurology and Stroke Centre, University Hospital Basel and University of Basel, Basel, Switzerland; ad Julius Centre for Health Sciences and Primary Care, University Medical Center Utrecht and Utrecht University, Utrecht, Netherlands; ae Department of Neurology and Neurosurgery, Utrecht Stroke Centre, University Medical Center Utrecht and Utrecht University, Utrecht, Netherlands; af Centre for Clinical Brain Sciences, School of Clinical Sciences, University of Edinburgh, Edinburgh, UK; ag Edinburgh Imaging, School of Clinical Sciences, University of Edinburgh, Edinburgh, UK; ah UK Dementia Institute at the University of Edinburgh, School of Clinical Sciences, University of Edinburgh, Edinburgh, UK; ai Lysholm Department of Neuroradiology and the Neuroradiological Academic Unit, Department of Brain Repair and Rehabilitation, UCL Institute of Neurology and the National Hospital for Neurology and Neurosurgery, London, UK; aj Liverpool Centre for Cardiovascular Science, University of Liverpool and Liverpool Heart and Chest Hospital, Liverpool, UK; ak Aalborg Thrombosis Research Unit, Department of Clinical Medicine, Aalborg University, Aalborg, Denmark; al Department of Diagnostic and Interventional Neuroradiology and Department of Neurology Inselspital, University Hospital Bern, University of Bern, Bern, Switzerland; am Department of Neurology, Sainte-Anne Hospital, Paris Descartes University, INSERM U1266, Paris, France; an Department of Neuroradiology, Sainte-Anne Hospital, Paris Descartes University, INSERM U1266, Paris, France; ao Peninsula Clinical School, Peninsula Health, Monash University, Melbourne, VIC, Australia; ap Stroke and Ageing Research Group, School of Clinical Sciences at Monash Health, Monash University, Melbourne, VIC, Australia; aq Department of Neurology, University Hospital of Würzburg, Josef-Schneider Strasse 11, Würzburg, Germany; ar Department of Imaging and Interventional Radiology, Prince of Wales Hospital, The Chinese University of Hong Kong, Ma Liu Shui, Hong Kong; as Department of Medicine and Therapeutics, Prince of Wales Hospital, The Chinese University of Hong Kong, Ma Liu Shui, Hong Kong; at Institute of Cardiovascular and Medical Science, University of Glasgow, Glasgow, UK; au The Neurovascular Research Unit and Health Research Board, Stroke Clinical Trials Network Ireland, University College Dublin, Dublin, Ireland; av Department of Geriatric and Stroke Medicine, Royal College of Surgeons in Ireland, Dublin, Ireland; aw Beaumont Hospital Dublin, Ireland; ax Department of Radiology and Nuclear Medicine, Maastricht University Medical Centre, Maastricht, Netherlands; ay Department of Neurology, CARIM School for Cardiovascular Diseases, Maastricht University Medical Centre, Maastricht, Netherlands; az Comprehensive Stroke Service, University College London Hospitals NHS Trust, London, UK; ba Department of Neurology, Donders Institute for Brain, Cognition and Behaviour, Donders Centre for Medical Neuroscience, Radboud University Medical Center, Nijmegen, Netherlands; bb Lucerne State Hospital; Switzerland Center for Neurology and Neurorehabilitation, Luzern, Switzerland; bc Department of Neurology, National Neuroscience Institute, Singapore, Singapore; bd University of Lille, Inserm, CHU de Lille, Degenerative and vascular cognitive disorders U1171, Lille, France; be Department of Psychiatry and Neuropsychology, School for Mental Health and Neuroscience, Maastricht University, Maastricht, Netherlands; bf Department of Neurology, The 2nd Affiliated Hospital of Zhejiang University, School of Medicine, Hangzhou, China; bg Memory Aging and Cognition Centre, National University Health System, Singapore, Singapore; bh Department of Neurology, Onze Lieve Vrouwe Gasthuis, Amsterdam, Netherlands; bi Neurology and Neurorehabilitation, University Department of Geriatric Medicine Felix Platter, University of Basel, Basel, Switzerland; bj Stroke Division, Florey Institute of Neuroscience and Mental Health, University of Melbourne, Melbourne, VIC, Australia; bk Department of Neurology, Austin Health, Melbourne, VIC, Australia; bl Department of Neurosciences, University Hospitals Leuven, Belgium; bm Department of Stroke Medicine, Imperial College London, London, UK; bn Department of Neurology, Heidelberg University Hospital, Heidelberg, Germany; bo Unit of Neuroradiology, Hospital Santa Creu i Sant Pau, Universitat Autonoma, Barcelona, Spain; bp Department of Radiology and Nuclear Medicine, Erasmus Medical Centre, University Medical Centre, Rotterdam, Netherlands

## Abstract

**Background:**

Cerebral microbleeds are a neuroimaging biomarker of stroke risk. A crucial clinical question is whether cerebral microbleeds indicate patients with recent ischaemic stroke or transient ischaemic attack in whom the rate of future intracranial haemorrhage is likely to exceed that of recurrent ischaemic stroke when treated with antithrombotic drugs. We therefore aimed to establish whether a large burden of cerebral microbleeds or particular anatomical patterns of cerebral microbleeds can identify ischaemic stroke or transient ischaemic attack patients at higher absolute risk of intracranial haemorrhage than ischaemic stroke.

**Methods:**

We did a pooled analysis of individual patient data from cohort studies in adults with recent ischaemic stroke or transient ischaemic attack. Cohorts were eligible for inclusion if they prospectively recruited adult participants with ischaemic stroke or transient ischaemic attack; included at least 50 participants; collected data on stroke events over at least 3 months follow-up; used an appropriate MRI sequence that is sensitive to magnetic susceptibility; and documented the number and anatomical distribution of cerebral microbleeds reliably using consensus criteria and validated scales. Our prespecified primary outcomes were a composite of any symptomatic intracranial haemorrhage or ischaemic stroke, symptomatic intracranial haemorrhage, and symptomatic ischaemic stroke. We registered this study with the PROSPERO international prospective register of systematic reviews, number CRD42016036602.

**Findings:**

Between Jan 1, 1996, and Dec 1, 2018, we identified 344 studies. After exclusions for ineligibility or declined requests for inclusion, 20 322 patients from 38 cohorts (over 35 225 patient-years of follow-up; median 1·34 years [IQR 0·19–2·44]) were included in our analyses. The adjusted hazard ratio [aHR] comparing patients with cerebral microbleeds to those without was 1·35 (95% CI 1·20–1·50) for the composite outcome of intracranial haemorrhage and ischaemic stroke; 2·45 (1·82–3·29) for intracranial haemorrhage and 1·23 (1·08–1·40) for ischaemic stroke. The aHR increased with increasing cerebral microbleed burden for intracranial haemorrhage but this effect was less marked for ischaemic stroke (for five or more cerebral microbleeds, aHR 4·55 [95% CI 3·08–6·72] for intracranial haemorrhage *vs* 1·47 [1·19–1·80] for ischaemic stroke; for ten or more cerebral microbleeds, aHR 5·52 [3·36–9·05] *vs* 1·43 [1·07–1·91]; and for ≥20 cerebral microbleeds, aHR 8·61 [4·69–15·81] *vs* 1·86 [1·23–2·82]). However, irrespective of cerebral microbleed anatomical distribution or burden, the rate of ischaemic stroke exceeded that of intracranial haemorrhage (for ten or more cerebral microbleeds, 64 ischaemic strokes [95% CI 48–84] per 1000 patient-years *vs* 27 intracranial haemorrhages [17–41] per 1000 patient-years; and for ≥20 cerebral microbleeds, 73 ischaemic strokes [46–108] per 1000 patient-years *vs* 39 intracranial haemorrhages [21–67] per 1000 patient-years).

**Interpretation:**

In patients with recent ischaemic stroke or transient ischaemic attack, cerebral microbleeds are associated with a greater relative hazard (aHR) for subsequent intracranial haemorrhage than for ischaemic stroke, but the absolute risk of ischaemic stroke is higher than that of intracranial haemorrhage, regardless of cerebral microbleed presence, antomical distribution, or burden.

**Funding:**

British Heart Foundation and UK Stroke Association.

## Introduction

A central challenge in stroke prevention after ischaemic stroke or transient ischaemic attack is to predict the risk of intracranial haemorrhage and to differentiate this from the risk of recurrent ischaemic stroke in patients treated with antithrombotic therapy—usually antiplatelet drugs or, in patients with atrial fibrillation, oral anticoagulants.^1^ Cerebral microbleeds are a radiological finding of small (<10 mm), hypointense (black), ovoid or rounded regions on T2*-weighted gradient-recalled echo (GRE) or susceptibility-weighted imaging (SWI).^2^ Cerebral microbleeds mostly correspond pathologically to haemosiderin-laden macrophages close to arterioles affected by small vessel diseases;^3^, ^4^ strictly lobar cerebral microbleeds suggest cerebral amyloid angiopathy (CAA), whereas deep patterns probably indicate arteriolosclerosis and mixed patterns probably indicate mixed pathologies.^5^, ^6^, ^7^, ^8^ Cerebral microbleeds might result from red blood cell leakage from arterioles and capillaries, raising clinical concerns that they herald an increased risk of potentially devastating intracranial haemorrhage, particularly in patients treated with antithrombotic drugs.^9^ However, cerebral microbleeds signal small vessel diseases that can also cause ischaemic stroke, and might result from non-haemorrhagic mechanisms.^10^, ^11^, ^12^, ^13^ In ischaemic stroke cohorts, cerebral microbleeds are associated with the risks of both subsequent intracranial haemorrhage and recurrent ischaemic stroke.^14^, ^15^, ^16^, ^17^, ^18^, ^19^, ^20^, ^21^, ^22^, ^23^, ^24^, ^25^, ^26^, ^27^, ^28^ As the number of cerebral microbleeds increases, the risk of intracranial haemorrhage seems to rise more steeply than that of ischaemic stroke, and having five or more cerebral microbleeds has been reported to be associated with similar absolute risks of intracranial haemorrhage and ischaemic stroke.^28^, ^29^

Because previous studies had small sample sizes and few intracranial haemorrhage outcome events, they could not reliably answer the important clinical question of whether many cerebral microbleeds, or patterns (distributions) of cerebral microbleeds, indicate a higher risk of intracranial haemorrhage than of recurrent ischaemic stroke. We established the Microbleeds International Collaborative Network^30^ to undertake large-scale pooled analyses of prospective observational cohort studies. We tested the hypothesis that a large burden of cerebral microbleeds, or their anatomical patterns, can identify ischaemic stroke or transient ischaemic attack patients at higher absolute risk of intracranial haemorrhage than ischaemic stroke.

**Research in context**

**Evidence before this study**

We searched Medline and EMBASE from Jan 1, 1996, to Dec 1, 2018 (search strategy: “cerebral adj2 micro*” OR “CMB” OR “microbleed.mp” AND [“stroke.mp” OR “stroke/” OR “intracerebral h?emorr*” OR “intracranial h?emorr*” OR “isch?emic stroke” OR “isch?emic infarct*”]) for studies in English that included patients with ischaemic stroke or transient ischaemic attack in whom the presence and anatomical distribution of cerebral microbleeds were measured at baseline, with at least 90 days of follow-up. An aggregate level meta-analysis (n=5068) showed that cerebral microbleeds were associated with both intracranial haemorrhage (risk ratio [RR] 3·8 [95% CI 3·5–11·4]) and ischaemic stroke (RR 1·8 [1·4–2·5]); this pooled analysis, and another study in two cohorts (one including 1003 mainly Chinese participants and the other including 1080 mainly white participants) reported that five or more cerebral microbleeds were associated with similar absolute risks of intracranial haemorrhage and ischaemic stroke. However, small sample sizes and few intracranial haemorrhage outcome events in previous studies did not provide enough statistical power and precision to establish whether a large cerebral microbleed burden or distribution pattern is associated with a higher absolute risk of intracranial haemorrhage than ischaemic stroke in patients with recent ischaemic stroke or transient ischaemic attack treated with antithrombotic drugs.

**Added value of this study**

Our pooled analysis of individual data from 20 322 patients shows that regardless of cerebral microbleed burden and distribution (ie, mixed, deep, or lobar), or the type of antithrombotic treatment received (oral anticoagulants or antiplatelet therapy), the absolute rate of ischaemic stroke is consistently substantially higher than that of intracranial haemorrhage. By contrast with previous studies, the large number of participants provided more precise estimates of stroke recurrence rates and risks, while inclusion of individual patient data allowed adjustment for potential confounding factors. Our study adds new data for patients with many (eg, ≥20) cerebral microbleeds, which cause the most clinical concern regarding intracranial bleeding.

**Implications of all the available evidence**

Although cerebral microbleeds can inform regarding the hazard for intracranial haemorrhage in patients with recent ischaemic stroke or transient ischaemic attack treated with antithrombotic drugs, the absolute risk of ischaemic stroke is much higher than that of intracranial haemorrhage, regardless of cerebral microbleed presence, burden, or pattern. The available evidence does not support witholding antithrombotic treatment because of cerebral microbleeds, but to definitively answer this question requires data from randomised controlled trials.

## Methods

### Study design

For this pooled analysis of individual patient data, we identified cohorts by searching Medline and EMBASE (search terms “cerebral adj2 micro*” OR “CMB” OR “microbleed.mp” AND “stroke.mp” OR “stroke/” OR “intracerebral h?emorr*” OR “intracranial h?emorr*” OR “isch?emic stroke” OR “isch?emic infarct*”), clinical trial databases (clinicaltrials.gov and strokecenter.org), and scientific meeting abstracts. We invited members of the METACOHORTS consortium;^31^ an international database of more than 90 studies of small vessel disease, including 660 000 patients. Two authors (DW and DJWe) independently did the search and reviewed all titles and abstracts; they also did an independent risk of bias assessment for all included studies. Cohorts were eligible for inclusion if they prospectively recruited adult participants with ischaemic stroke or transient ischaemic attack; included at least 50 participants; collected data on stroke events over at least 3 months follow-up; used an appropriate MRI sequence that is sensitive to magnetic susceptibility (GRE or SWI); and documented the number and anatomical distribution of cerebral microbleeds reliably using consensus criteria and validated scales. Each patient was only included in one cohort. We assessed all studies for risk of bias (including selection bias) and quality using the Cochrane Collaboration tool.^32^ All cohorts obtained ethical approval as required by local regulations to allow data sharing. All data reviewed by the co-ordinating centre was fully anonymised. The project was approved by the Health Research Authority of the UK (REC reference: 8/HRA/0188). The Microbleeds International Collaborative Network protocol and statistical analysis plan were registered with PROSPERO on April 5, 2016 (CRD42016036602).

### Outcomes

Our prespecified primary outcomes were a composite of any symptomatic intracranial haemorrhage (confirmed radiologically, including subdural, extradural, and subarachnoid haemorrhage, and excluding intracranial haemorrhages attributed to intravenous thrombolysis or trauma) or ischaemic stroke (acute or subacute neurological symptoms lasting >24 h and attributed to cerebral ischaemia, diagnosed clinically, with or without radiological confirmation); symptomatic intracranial haemorrhage; and symptomatic ischaemic stroke. Secondary outcome events were death (all cause) and vascular death. All events were adjudicated according to individual cohort protocols.

### Statistical analysis

As per our prespecified protocol, a single dataset was created by combining individual participant data from the 38 cohorts. We compared baseline demographic and risk factor profiles between patients with and without cerebral microbleeds and between patients with and without outcome events using the Mann-Whitney test if not normally distributed or the *t* test if normally distributed; we compared categorical variables between groups with the χ^2^ test or Fisher's exact test. We censored patients at the last available follow-up (truncated to 5 years) or at the time of the prespecified outcome event. When a patient had multiple events of the same type, we censored follow-up at the first event. We calculated absolute event rates per 1000 patient-years for primary outcomes in patients with and without cerebral microbleeds. We assessed the proportional hazards assumption through visual inspection of (log–log) plots of log cumulative hazard against time and tested for a non-zero slope in a regression of scaled Schoenfeld residuals against time. We calculated univariate Kaplan-Meier survival probabilities in patients with and without cerebral microbleeds to estimate event rates and used the log-rank test to compare groups. We did multivariable Cox regression adjusting for the following prognostic and confounding variables (selected by consensus based on availability, biological plausibility, and known associations with cerebral microbleeds and outcomes): age, sex, presentation with transient ischaemic attack or ischaemic stroke, history of hypertension, previous stroke, known atrial fibrillation, antithrombotic use after index event, and type of MRI sequence used to detect cerebral microbleeds (T2*-weighted GRE or SWI). We investigated the effect of predefined cerebral microbleed burden categories (one, two to four, five or more, ten or more, and 20 or more). When investigating cerebral microbleed distribution, we adjusted for number of cerebral microbleeds. We added a shared frailty term^33^ to account for patients being nested in individual studies (thus potentially having correlated data). We performed subanalyses for patients treated with oral anticoagulants and antiplatelet drugs and added interaction terms between antithrombotic therapy and presence of cerebral microbleeds. We categorised ethnicity (when available) as white or Asian (Japanese, Chinese, Malays, Indian, Pakistani, or Korean) to investigate the interaction between ethnicity and cerebral microbleed presence. We performed two prespecified sensitivity analyses: the first exploring time-varying risks within the Cox model to investigate later events (beyond the first year) accounting for death as a competing risk (using the Fine-Gray subdistribution hazard model), calculating subdistribution hazard ratios (sHRs); and the second, a two-stage individual-patient meta-analysis to quantify between-study heterogeneity using the inverse-variance method (which fits a separate survival model for each cohort then pools and displays estimates in a forest plot). We did three post-hoc analyses as follows: (1) we added white matter hyperintensities (another common marker of cerebral small vessel disease, rated using the Fazekas scale^34^ and considered severe if rated two or greater in the periventricular of deep white matter) into our multivariable model; (2) we included only intracerebral haemorrhage, convexity subarachnoid haemorrhage, and subdural haemorrhage, because these bleeding events are the most likely to be associated with cerebral microbleeds; and (3) we investigated the interaction between cerebral microbleeds and age (<80 years or ≥80 years). In sensitivity analyses, if data for a variable of interest was not sufficiently available in a cohort, the cohort was excluded. We did all statistical analysis using STATA, version 15.

### Role of the funding source

The funder of the study had no role in the study design, data collection, data analysis, or data interpretation, or writing of the report. The corresponding author had full access to all the data in the study and had final responsibility for the decision to submit for publication.

## Results

**Figure 1:**
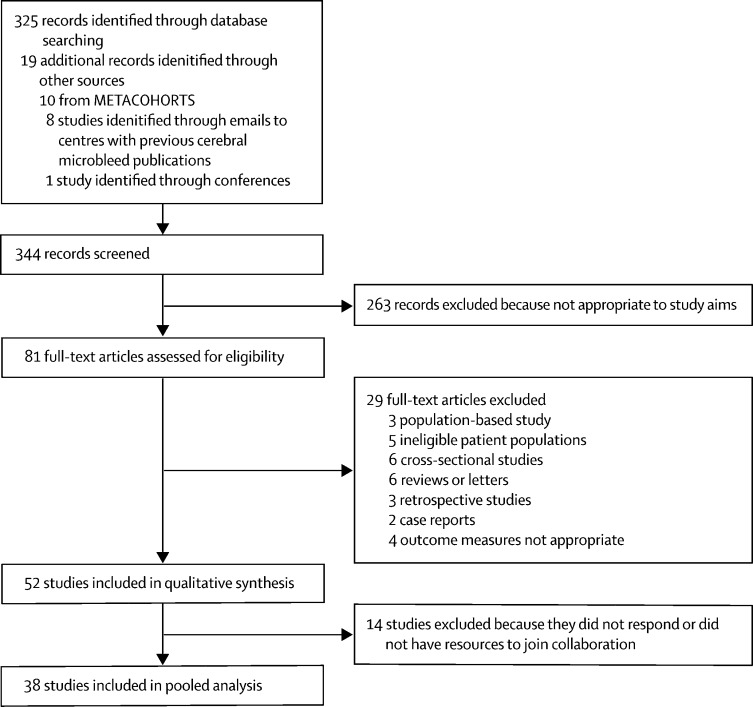
Study selection profile

Between Jan 1, 1996, and Dec 1, 2018, we identified and screened 344 records (325 from database search and 19 from other sources; figure 1). 263 records were excluded because they were not full-text articles, and then a further 29 full-text articles were excluded because they did not meet study inclusion criteria. The remaining 52 studies were included in our qualitative analyses, but 14 of these were excluded from the meta-analysis because they did not respond to requests for individual patient data or declined to join the collaboration (reasons included a lack of resources or because of data sharing policies). From the 38 remaining cohorts (23 published and 15 unpublished studies), we included 20 322 participants (table 1). Although more than half of participants and outcome events came from the six largest cohorts, no major risk of bias was detected for any included cohort (appendix). The mean age of participants was 70 years (SD 13); 8593 (42%) of the 20 322 were women. Cerebral microbleeds were present in 5649 (28%) patients (appendix), including 2415 (12%) with one cerebral microbleed, 1990 (10%) with two to four cerebral microbleeds, and 1244 (6%) with five or more cerebral microbleeds. Over the 35 225 patient-years of follow-up (median 1·34 years [IQR 0·19–2·44]), 1474 composite events occurred: 189 intracranial haemorrhages; 1113 ischaemic strokes; and 172 composite events of unknown type from one cohort of 3355 participants, which did not subclassify composite outcomes as intracranial haemorrhage or ischaemic stroke. Characteristics between patients with and without events are in the appendix. Visual assessment of the log-log plots and the results of testing the Schoenfeld residuals suggest that the proportional hazards assumption was not violated in any of the following analyses.

**Table 1:** Demographics, risk factors, and outcome events for each cohort

	**Total partici-pants**	**Taking oral anticoagu-lants**	**Transient ischaemic attack**	**Mean age (SD), years**	**Proportion women**	**Hyper-tension**	**Atrial fibrillation**	**Previous stroke**	**Ischaemic heart disease**	**Any cerebral microbleed**	**Susceptibility-weighted imaging**	**Median follow-up, days (IQR)**	**Patients with composite events**	**Participants with intracranial haemorrhage events**	**Participants with ischaemic stroke events**
CROMIS-2^27^	1490	1436 (96%)	238 (16%)	76 (10)	631 (42%)	930 (63%)	1490 (100%)	148 (10%)	243 (16%)	311 (21%)	0	774 (705–974)	70 (5%)	14 (1%)	56 (4%)
HBS	660	114 (17%)	60 (9%)	69 (15)	289 (44%)	498 (75%)	194 (29%)	156 (24%)	138 (21%)	98 (15%)	2 (<1%)	90 (90–90)	4 (1%)	0	4 (1%)
IPAAC-Warfarin^35^	182	173 (95%)	27 (15%)	73 (9)	84 (46%)	158 (87%)	179 (98%)	36 (20%)	17 (9%)	68 (37%)	182 (100%)	738 (191–812)	7 (4%)	1 (1%)	6 (3%)
Bern^36^	392	74 (19%)	0	68 (14)	169 (43%)	249 (64%)	142 (46%)	59 (15%)	69 (18%)	90 (23%)	392 (100%)	93 (5–106)	16 (4%)	0	16 (4%)
CU-STRIDE^37^	536	24 (4%)	81 (15%)	67 (11)	227 (42%)	373 (70%)	38 (7%)	80 (15%)	35 (7%)	124 (23%)	238 (44%)	524 (472–557)	17 (3%)	2 (<1%)	15 (3%)
TABASCO^38^	436	33 (8%)	213 (28%)	67 (9)	184 (43%)	250 (59%)	33 (8%)	0	60 (14%)	64 (15%)	0	1825 (1164–1825)	57 (13%)	0	57 (13%)
Graz	460	78 (17%)	48 (10%)	67 (13)	179 (39%)	359 (78%)	115 (25%)	102 (22%)	95 (21%)	88 (19%)	0	117 (87–973)	65 (14%)	13 (3%)	54 (12%)
PERFORM-MRI^39^	1056	0	127 (12%)	68 (8)	370 (35%)	887 (84%)	16 (2%)	120 (11%)	69 (7%)	381 (36%)	0	774 (701–1042)	104 (10%)	10 (1%)	94 (9%)
PARISK^40^	228	0	127 (56%)	71 (9)	67 (29%)	156 (68%)	0	66 (29%)	50 (22%)	61 (27%)	0	786 (757–819)	10 (4%)	0	10 (4%)
SAMURAI NVAF^41^	1103	1039 (94%)	45 (4%)	78 (10)	480 (44%)	1027 (93%)	1103 (100%)	246 (22%)	101 (9%)	265 (24%)	817 (74%)	723 (758–818)	82 (7%)	10 (1%)	72 (7%)
RUNDMC^42^	179	19 (11%)	89 (50%)	65 (9)	63 (35%)	145 (81%)	18 (10%)	47 (26%)	31 (17%)	35 (20%)	0	1825 (1825–1825)	25 (14%)	2 (1%)	23 (13%)
Wuerzburg	358	122 (34%)	77 (22%)	71 (13)	158 (44%)	287 (80%)	105 (29%)	91 (25%)	38 (11%)	87 (24%)	160 (45%)	95 (89–103)	22 (6%)	1 (<1%)	21 (6%)
Monash Stroke^43^	359	356 (99%)	52 (15%)	75 (11)	173 (48%)	285 (79%)	359 (100%)	101 (28%)	122 (34%)	154 (43%)	339 (94%)	530 (280–898)	14 (4%)	7 (2%)	9 (3%)
Basel TIA^18^	192	33 (17%)	192 (100%)	69 (13)	73 (38%)	137 (71%)	26 (14%)	13 (7%)	38 (20%)	21 (11%)	0	90 (90–90)	26 (14%)	0	26 (14%)
Yonsei^44^	504	487 (97%)	28 (6%)	70 (11)	288 (57%)	392 (78%)	504 (100%)	101 (20%)	109 (22%)	155 (31%)	0	849 (393–1398)	56 (11%)	7 (1%)	49 (10%)
SNUBH Stroke Cohort^45^, ^46^	3355	625 (19%)	368 (11%)	67 (13)	1347 (40%)	2324 (69%)	630 (19%)	487 (15%)	284 (8%)	1166 (35%)	1 (<1%)	355 (340–365)	172 (5%)	NA	NA
BIOSTROKE/TIA^47^	260	73 (28%)	160 (62%)	68 (13)	95 (37%)	150 (59%)	77 (31%)	21 (8%)	57 (22%)	24 (9%)	0	90 (90–365)	14 (5%)	0	14 (5%)
Kushiro City^48^	784	63 (8%)	0	72 (11)	330 (42%)	498 (64%)	104 (13%)	142 (18%)	89 (11%)	320 (41%)	0	1008 (105–1825)	139 (18%)	22 (3%)	119 (15%)
Soo^49^	81	81 (100%)	16 (20%)	72 (9)	40 (49%)	56 (69%)	81 (100%)	25 (31%)	8 (10%)	24 (30%)	71 (88%)	737 (641–794)	8 (10%)	3 (4%)	5 (6%)
CASPER^50^	135	18 (13%)	0	66 (11)	39 (29%)	96 (71%)	13 (10%)	10 (7%)	29 (21%)	79 (59%)	135 (100%)	453 (444–465)	3 (2%)	0	3 (2%)
HERO^51^	937	933 (>99%)	122 (13%)	78 (7)	488 (52%)	694 (74%)	468 (50%)*	248 (27%)	146 (16%)	248 (26%)	0	737 (641–794)	49 (5%)	18 (2%)	32 (3%)
HAGAKURE	426	157 (37%)	35 (8%)	74 (13)	174 (41%)	320 (76%)	135 (32%)	76 (18%)	45 (11%)	158 (37%)	39 (9%)	748 (350–1040)	34 (8%)	9 (2%)	25 (6%)
Leuven^14^	487	133 (27%)	133 (27%)	72 (9)	192 (39%)	313 (64%)	103 (21%)	61 (13%)	112 (23%)	129 (26%)	0	804 (686–968)	36 (7%)	4 (1%)	32 (7%)
NOACISP	306	286 (93%)	30 (10%)	73 (19)	139 (45%)	240 (79%)	306 (100%)	60 (20%)	83 (27%)	87 (28%)	300 (98%)	735 (417–836)	28 (9%)	10 (3%)	19 (6%)
Min Lou^52^	126	14 (11%)	0	65 (13)	46 (37%)	94 (75%)	25 (20%)	18 (14%)	4 (3%)	42 (33%)	126 (100%)	92 (87–218)	2 (2%)	0	2 (2%)
MICRO^21^	397	40 (10%)	362 (91%)	65 (12)	165 (42%)	218 (55%)	30 (8%)	35 (9%)	24 (6%)	72 (18%)	0	1212 (579–1825)	30 (8%)	11 (3%)	21 (5%)
Orken^53^	454	454 (100%)	20 (4%)	72 (12)	233 (51%)	258 (79%)	296 (65%)	123 (27%)	79 (32%)†	134 (30%)	250 (55%)	575 (228–1825)	11 (2%)	3 (1%)	8 (2%)
CATCH^54^	416	67 (16%)	173 (42%)	67 (14)	164 (39%)	226 (54%)	27 (6%)	0	NA	65 (16%)	0	88 (80–100)	14 (3%)	1 (<1%)	13 (3%)
MSS2^55^	263	24 (9%)	0	67 (12)	109 (41%)	190 (72%)	25 (10%)	32 (12%)	53 (20%)	44 (17%)	251 (95%)	368 (253–403)	31 (12%)	0	31 (12%)
Sainte-Anne (Paris)	385	302 (78%)	0	80 (11)	204 (53%)	277 (72%)	358 (100%)	61 (16%)	72 (19%)	99 (26%)	0	440 (163–733)	25 (6%)	5 (1%)	23 (6%)
STROKDEM	181	48 (27%)	0	64 (13)	69 (38%)	100 (55%)	12 (7%)	20 (11%)	17 (9%)	24 (13%)	0	1150 (420–1820)	17 (9%)	0	17 (9%)
Singapore (Chen)	45	15 (33%)	0	67 (10)	13 (29%)	34 (76%)	11 (24%)	3 (7%)	4 (9%)	25 (56%)	45 (100%)	1057 (703–1199)	6 (13%)	0	6 (13%)
FUTURE Study	19	0	7 (37%)	44 (6)	10 (53%)	8 (42%)	0	0	0	1 (5%)	19 (100%)	164 (131–242)	4 (21%)	0	4 (21%)
Heidelberg^56^	650	119 (18%)	109 (17%)	64 (14)	240 (37%)	496 (76%)	115 (18%)	107 (17%)	NA	155 (24%)	650 (100%)	1534 (1271–1825)	34 (5%)	4 (1%)	30 (5%)
NNI	184	32 (17%)	0	58 (11)	57 (31%)	143 (78%)	28 (15%)	27 (15%)	NA	50 (27%)	0	251 (86–477)	0	0	0
OXVASC^29^	1080	118 (11%)	572 (52%)	68 (14)	514 (48%)	588 (55%)	167 (15%)	201 (19%)	146 (13%)	157 (15%)	0	1271 (681–1825)	90 (8%)	11 (1%)	79 (7%)
HKU^29^	1003	104 (10%)	0	69 (12)	402 (40%)	657 (66%)	130 (13%)	116 (12%)	92 (9%)	450 (45%)	1003 (100%)	1005 (599–1549)	112 (11%)	20 (2%)	92 (9%)
SIGNaL	213	43 (20%)	22 (10%)	72 (14)	88 (41%)	152 (71%)	67 (32%)	60 (28%)	49 (23%)	94 (44%)	144 (68%)	225 (202–249)	27 (13%)	1 (<1%)	26 (12%)
Total	20 322	7737/20 319 (38%)	3443/20 311 (17%)	70 (13)	8593/20 314 (42%)	14 365/20 271 (71%)	7557/20 207 (37%)	3299/20 290 (16%)	2608/18 842 (14%)	5649/20 322 (28%)	5164/20 284 (25%)	534 (243–928)	1461/20 322 (7%)	189/16 967 (1%)	1113/16 967 (7%)

Data are n (%) or n/N (%) unless otherwise stated. Studies without references are unpublished. FUTURE study=Follow-Up of Transient ischemic attack and stroke patients and Unelucidated Risk factor Evaluation study. HAGAKURE=Hypertension, Amyloid, and aGe Associated Kaleidoscopic brain lesions on CT/MRI Undertaken with stroke REgistry. HBS=Heart Brain Interactions Study. NNI=National Neuroscience Institute, Singapore. NOACISP=Novel Oral Anticoagulants in Stroke Patients, Basel; NCT02353585. SIGNaL=Stroke Investigation in North and Central London. STROKDEM=Study of Factors Influencing Post-stroke Dementia.

* Denominator for this result is 932.

† Denominator for this result is 250.

**Figure 2:**
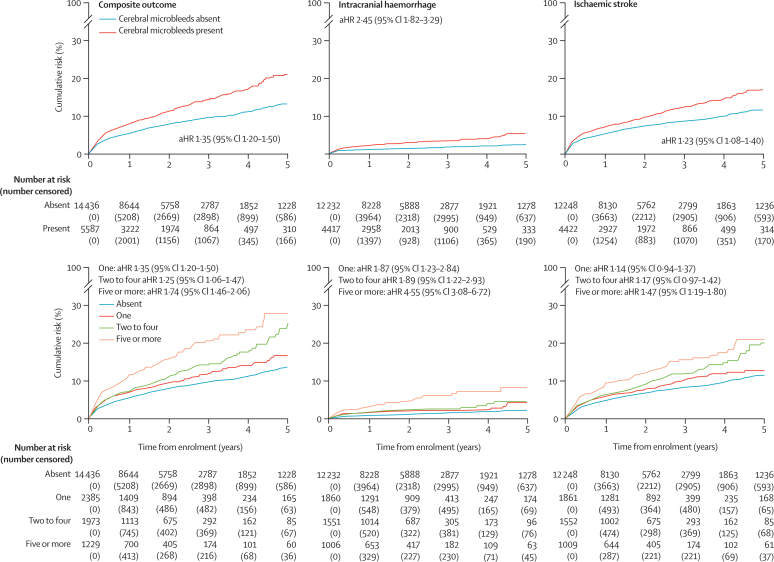
Kaplan-Meier estimates for the primary outcomes in all patients (n=20 322)

The composite outcome of any intracranial haemorrhage or ischaemic stroke (aHR 1·35 [95% CI 1·20–1·50], p<0·0001; log-rank test), symptomatic intracranial haemorrhage (2·45 [1·82–3·29], p<0·0001), and symptomatic ischaemic stroke (1·23 [1·08–1·40], p<0·0001) were more frequent in patients with cerebral microbleeds than those without (figure 2; appendix).

The incidence of all composite events in patients with any cerebral microbleed was 59 per 1000 patient-years (95% CI 54–64) compared with 35 per 1000 patient-years (33–38) in those without cerebral microbleeds, an absolute increased incidence of 24 per 1000 patient-years (21–26; table 2). The aHR for a composite event became larger with increased cerebral microbleed burden (figure 2, table 2; p_trend_<0·0001). aHRs were similar across different cerebral microbleed anatomical distributions (table 2).

**Table 2:** Rate and risk of outcome events according to number (burden) and anatomical distribution of baseline cerebral microbleeds in all patients (n=20 322)

		**Composite of intracranial haemorrhage and ischaemic stroke (n=19 816 for multivariable model)**			**Symptomatic intracranial haemorrhage (n=16 447 for multivariable model)**			**Symptomatic ischaemic stroke (n=16 464 for multivariable model)**		
		Rate, per 1000 patient-years*	Absolute rate increase, per 1000 patient-years	Adjusted hazard ratio	Rate, per 1000 patient-years	Absolute rate increase, per 1000 patient-years	Adjusted hazard ratio	Rate, per 1000 patient-years	Absolute rate increase, per 1000 patient-years	Adjusted hazard ratio
None		35 (33–38)	..	1 (ref)	4 (3–5)	..	1 (ref)	30 (28–33)	..	1 (ref)
Any		59 (54–64)	24 (21–26)	1·35 (1·20–1·50)	12 (10–14)	8 (7–9)	2·45 (1·82–3·29)	46 (42–51)	16 (14–18)	1·23 (1·08–1·40)
One		46 (40–53)	11 (7–15)	1·21 (1·03–1·42)	8 (5–12)	4 (2–7)	1·87 (1·23–2·84)	37 (31–44)	7 (3–11)	1·14 (0·94–1·37)
Number										
	Two to four	58 (50–67)	23 (17–29)	1·25 (1·06–1·47)	9 (6–14)	5 (3–9)	1·89 (1·22–2·93)	48 (40–56)	18 (12–23)	1·17 (0·97–1·42)
	Five or more†	85 (73–99)	50 (40–61)	1·74 (1·46–2·06)	23 (16–31)	19 (13–26)	4·55 (3·08–6·72)	64 (53–77)	34 (25–43)	1·47 (1·19–1·80)
	Ten or more†	91 (73–113)	56 (40–75)	1·82 (1·44–2·29)	27 (17–41)	23 (14–36)	5·52 (3·36–9·05)	64 (48–84)	34 (20–51)	1·43 (1·07–1·91)
	20 or more†	118 (86–160)	83 (53–122)	2·61 (1·90–3·57)	39 (21–67)	35 (18–62)	8·61 (4·69–15·81)	73 (46–108)	43 (18–75)	1·86 (1·23–2·82)
Anatomical distribution										
	Mixed	80 (68–94)	45 (35–56)	1·28 (1·06–1·54)	20 (14–28)	16 (11–23)	2·38 (1·55–3·65)	60 (49–73)	30 (21–40)	1·12 (0·88–1·41)
	Deep	73 (65–82)	38 (32–44)	1·29 (1·12–1·48)	17 (13–22)	13 (10–17)	2·57 (1·78–3·70)	57 (49–66)	27 (21–33)	1·14 (0·96–1·36)
	Lobar	60 (53–67)	25 (20–29)	1·22 (1·06–1·41)	13 (9–16)	9 (6–9)	1·87 (1·29–2·71)	48 (42–56)	18 (14–23)	1·17 (0·99–1·40)
	Probable cerebral amyloid angiopathy	55 (40–73)	20 (7–35)	1·21 (0·90–1·64)	9 (4–18)	5 (1–13)	1·29 (0·60–2·77)	48 (34–66)	18 (6–33)	1·31 (0·94–1·83)

Ranges in brackets are 95% CIs. Cerebral microbleed location hazard ratios are versus patients without cerebral microbleeds in each location and are adjusted for cerebral microbleed number and our prespecified variables.

* Number of patients and time at risk are shown in the appendix.

† Overlapping categories.

189 patients had a symptomatic intracranial haemorrhage over 32 847 patient-years of follow-up (151 intracerebral haemorrhages, 31 subdural haemorrhages, eight subarachnoid haemorrhages [four of which were cortical], and three extradural haemorrhages; four patients had more than one type of intracranial haemorrhage). The incidence of intracranial haemorrhage was 12 per 1000 patient-years (95% CI 10–14) in those with cerebral microbleeds compared with 4 per 1000 patient-years (3–5) in those without cerebral microbleeds, an absolute increased incidence of 8 per 1000 patient-years (7–9; table 2). The rate of intracranial haemorrhage increased with increasing cerebral microbleed burden, but was consistently lower than the rate of ischaemic stroke (table 2). The aHR for symptomatic intracranial haemorrhage was 2·45 (95% CI 1·82–3·29) for patients with cerebral microbleeds versus those without, and became larger with increased cerebral microbleed burden (p_trend_ <0·0001; figure 2; table 2); aHRs did not significantly differ between different cerebral microbleed anatomical distributions. Patients with multiple strictly lobar cerebral microbleeds (fulfilling the Boston criteria^5^ for probable CAA) did not have a significantly higher aHR for symptomatic intracranial haemorrhage than those without multiple strictly lobar cerebral microbleeds (1·29 [95% CI 0·60–2·77]; table 2). No interaction was detected between cerebral microbleeds and antiplatelet medication (p_interaction_=0·358), oral anticoagulants (p_interaction_=0·717), or combined oral anticoagulants and antiplatelet medication (p_interaction_=0·163) for intracranial haemorrhage risk.

1113 patients had a symptomatic ischaemic stroke over 32 293 patient-years of follow-up. The incidence of symptomatic ischaemic stroke in patients with cerebral microbleeds was 46 per 1000 patient-years (95% CI 42–51) compared with 30 per 1000 patient-years (28–33) in those without, with an absolute increased incidence of 16 per 1000 patient-years (14–18; table 2). The rate of ischaemic stroke became greater with an increasing burden of cerebral microbleeds, and for each burden category substantially exceeded the rate of intracranial haemorrhage (table 2). The aHR for symptomatic ischaemic stroke was 1·23 (95% CI 1·08–1·40) for patients with cerebral microbleeds versus those without, and the aHR became larger with increasing cerebral microbleed burden (p_trend_=0·0053; figure 2; table 2). Cerebral microbleed anotomical distribution had little effect on ischaemic stroke risk (table 2). No interaction was detected between cerebral microbleeds and antiplatelet medication (p_interaction_=0·943) or oral anticoagulants (p_interaction_=0·408) for ischaemic stroke risk, but there was weak evidence for an interaction between cerebral microbleeds and combined use of oral anticoagulants and antiplatelet medication (p_interaction_=0·047).

There were 2148 deaths, 484 of which were due to vascular causes. In multivariable analyses, cerebral microbleed presence was not associated with all-cause death (aHR 1·03 [95% CI 0·94–1·12]) or vascular death (aHR 0·97 [0·79–1·19]). No interaction was detected between cerebral microbleeds and ethnicity (n=15 123; 6743 white and 8380 Asian) for the risks of the composite outcome of intracranial haemorrhage or ischaemic stroke (p_interaction_=0·707); intracranial haemorrhage (p_interaction_=0·537); or ischaemic stroke (p_interaction_=0·654). No interaction was detected between cerebral microbleed and older age (4376 patients older than 80 years) for the risk of the composite outcome (p_interaction_=0·538); intracranial haemorrhage (p_interaction_=0·219); or ischaemic stroke (p_interaction_=0·286).

Using a two-stage meta-analysis, the estimated risks associated with cerebral microbleed presence were consistent with our main model for the composite outcome (heterogeneity [*I*^2^=31·7%]; intracranial haemorrhage [*I*^2^=0%]; and ischaemic stroke [^2^=24·2%]; appendix).

23 cohorts, including 10 235 patients, provided ratings for white matter hyperintensities, which were moderate to severe (Fazekas grade ≥2) in 3105 (30%) patients. Including white matter hyperintensities in multivariable models did not substantially change the aHR associated with the presence of cerebral microbleeds for the composite outcome (aHR 1.30 [95% CI 1.12–1.52]); intracranial haemorrhage (aHR 2.44 [1.68–3.53]); or for ischaemic stroke (aHR 1.16 [0.98–1.37]).

In our sensitivity analysis including only intracerebral, convexity subarachnoid, and subdural intracranial haemorrhages, 183 patients had a symptomatic intracranial haemorrhage over 32 847 patient-years of follow-up. The aHR for symptomatic intracranial haemorrhage was 2·59 (95% CI 1·91–3·50) for patients with cerebral microbleeds versus patients without, and became larger with increasing burden. Compared with no cerebral microbleeds, aHRs were 1·92 (95% CI 1·25–2·94) for one cerebral microbleed; 2·02 (1·30–3·16) for two to four cerebral microbleeds; 4·88 (3·29–7·25) for five or more cerebral microbleeds; 5·87 (3·56–9·66) for ten or more cerebral microbleeds; and 9·32 (5·06–17·16) for 20 or more cerebral microbleeds. These results are consistent with our primary findings.

There were 102 symptomatic intracranial haemorrhages over 12 794 patient-years of follow-up within the first year, and 87 over 31 059 patient-years of follow-up after the first year. In patients with cerebral microbleeds, the rate of intracranial haemorrhage was 18 per 1000 patient-years (95% CI 14–23) within the first year, and 5 per 1000 patient-years (3–6) after the first year.

696 ischaemic strokes were recorded over 12 873 patient-years of follow-up within the first year and 417 symptomatic ischaemic strokes during 30 447 patient-years of follow-up after the first year. In patients with cerebral microbleeds, the rate of symptomatic ischaemic stroke within the first year was 70 (95% CI 62–80), then 18 (15–21) after the first year.

Accounting for death as a competing risk, we found no evidence for a change in risk over time associated with cerebral microbleed presence for intracranial haemorrhage (sHR 4·96 [95% CI 3·18–7·74] at day 0 *vs* 4·81 [3·15–7·35] after 1 year) or ischaemic stroke (sHR 1·46 [1·23–1·73] at day 0 *vs* 1·49 [1·27–1·75] after 1 year).

In those treated with oral anticoagulants after their index ischaemic stroke or transient ischaemic attack (n=7737; vitamin K antagonist=5253, non-vitamin K oral anticoagulant=2484), 91 intracranial haemorrhages occurred over 13 942 patient-years of follow-up, and 384 ischaemic strokes occurred over 13 737 patient-years of follow-up. For patients with cerebral microbleeds, the rate of intracranial haemorrhage was 12 per 1000 patient-years (95% CI 9–16); the rate of ischaemic stroke was 32 per 1000 patient-years (26–39; table 3). The rate of ischaemic stroke was much higher than that of intracranial haemorrhage for all cerebral microbleed burden and anatomical distribution categories; the aHR for intracranial haemorrhage for patients with cerebral microbleeds (*vs* those without) rose more steeply than that of ischaemic stroke with increasing cerebral microbleed burden. Mixed and deep cerebral microbleed distributions had similar aHRs for intracranial haemorrhage, but patients with lobar cerebral microbleeds had a lower risk of intracranial haemorrhage (table 3). Cerebral microbleeds were not significantly associated with ischaemic stroke risk. We found no evidence of an interaction between oral anticoagulants type (vitamin K antagonist *vs* direct oral anticoagulant) and cerebral microbleed presence for intracranial haemorrhage (p_interaction_=0·4) or ischaemic stroke (p_interaction_=0·61).

**Table 3:** Rate and risk of outcome events according to baseline cerebral microbleeds in patients treated with oral anticoagulants with or without antiplatelet drugs (n=7737)

		**Composite of intracranial haemorrhage and ischaemic stroke (n=7582 for multivariable model)**			**Symptomatic intracranial haemorrhage (n=6942 for multivariable model)**			**Symptomatic ischaemic stroke (n=6958 in multivariable models)**		
		Rate, per 1000 patient-years*	Absolute rate increase, per 1000 patient-years	Adjusted hazard ratio	Rate, per 1000 patient-years	Absolute rate increase, per 1000 patient-years	Adjusted hazard ratio	Rate, per 1000 patient-years	Absolute rate increase, per 1000 patient-years	Adjusted hazard ratio
None		31 (28 to 35)	..	1 (ref)	5 (3 to 6)	..	1 (ref)	27 (23 to 30)	..	1 (ref)
Any		46 (39 to 53)	15 (11 to 18)	1·30 (1·07 to 1·57)	12 (9 to 16)	7 (6 to 10)	2·49 (1·64 to 3·79)	32 (26 to 39)	5 (3 to 9)	1·07 (0·86 to 1·35)
One		38 (30 to 49)	7 (2 to 14)	1·19 (0·91 to 1·56)	10 (6 to 17)	5 (3 to 11)	2·15 (1·23 to 3·75)	26 (19 to 35)	−1 (−4 to 5)	0·96 (0·69 to 1·33)
Number										
	Two to four	47 (36 to 60)	16 (8 to 25)	1·23 (0·93 to 1·62)	11 (6 to 19)	6 (3 to 13)	2·22 (1·21 to 4·06)	36 (26 to 48)	11 (3 to 18)	1·10 (0·80 to 1·52)
	Five or more†	62 (45 to 84)	31 (17 to 49)	1·69 (1·22 to 2·35)	20 (11 to 34)	15 (8 to 28)	3·91 (2·08 to 7·34)	40 (26 to 59)	13 (3 to 29)	1·27 (0·84 to 1·91)
	Ten or more†	75 (46 to 116)	44 (18 to 81)	2·15 (1·35 to 3·43)	23 (8 to 50)	18 (5 to 44)	4·63 (1·92 to 11·22)	46 (24 to 81)	19 (1 to 51)	1·52 (0·84 to 2·67)
Anatomical distribution										
	Mixed	58 (42 to 77)	27 (14 to 42)	1·43 (1·02 to 2·00)	15 (7 to 26)	10 (4 to 20)	2·21 (1·09 to 4·47)	42 (29 to 60)	15 (6 to 30)	1·28 (0·85 to 1·94)
	Deep	52 (42 to 63)	21 (14 to 28)	1·43 (1·11 to 1·84)	14 (9 to 21)	9 (6 to 15)	2·71 (1·61 to 4·59)	35 (27 to 46)	8 (4 to 16)	1·16 (0·85 to 1·59)
	Lobar	41 (32 to 51)	10 (4 to 16)	1·13 (0·87 to 1·47)	10 (6 to 16)	5 (3 to 10)	1·63 (0·94 to 2·83)	29 (22 to 38)	2 (−1 to 8)	1·00 (0·73 to 1·38)
	Probable cerebral amyloid angiopathy	27 (13 to 47)	−4 (−15 to 12)	0·76 (0·41 to 1·39)	10 (3 to 25)	5 (0 to 19)	1·29 (0·47 to 3·57)	17 (7 to 35)	−10 (−16 to 5)	0·64 (0·30 to 1·37)

Ranges in brackets are 95% CIs. Cerebral microbleed location hazard ratios are versus patients without cerebral microbleeds in each location and are adjusted for cerebral microbleed number and our prespecified variables.

* Number of patients and time at risk are shown in the appendix.

† Overlapping categories.

In patients treated with antiplatelet drugs only (n=11 520), 93 intracranial haemorrhages occurred over 18 059 patient-years of follow-up and 664 ischaemic strokes occurred over 17 731 patient-years of follow-up. The rate of ischaemic stroke remained higher than that of intracranial haemorrhage for all cerebral microbleed burden and anatomical distribution categories (appendix); aHRs for intracranial haemorrhage and ischaemic stroke in patients with versus without cerebral microbleeds were similar to those in the full cohort, with little variation according to cerebral microbleed anatomical distribution (appendix).

Compared with patients who received antithrombotic treatment (oral anticoagulants or antiplatelets), those not treated with antithrombotic drugs (n=1065) were older (mean age 72 years [SD 14] for those not treated with antithrombotic drugs *vs* 70 years [SD 13] for those treated with antithrombotic drugs), a greater proportion were women (46% *vs* 42%), more had ischaemic stroke (91% *vs* 83%), more had a previous intracranial haemorrhage (6% *vs* 2%), more had atrial fibrillation (44% *vs* 37%), fewer had been taking regular antiplatelet drugs before the qualifying event (27% *vs* 34%), and more had been taking regular oral anticoagulants before the qualifying event (13% *vs* 8%). No difference in the prevalence of cerebral microbleeds was observed based on receiving antithrombotic treatment (29% *vs* 28%). In those not treated with any antithrombotic drugs, five had intracranial haemorrhages over 846 patient-years and 65 had ischaemic strokes over 825 patient-years. The aHRs associated with cerebral microbleed presence were 1·10 (95% CI 0·17–7·34) for intracranial haemorrhage and 1·51 (0·87–2·65) for ischaemic stroke.

## Discussion

Our large-scale pooled analysis of individual patient data confirms that, in patients with recent ischaemic stroke or transient ischaemic attack treated with antithrombotic drugs, cerebral microbleeds are associated with the subsequent risks of symptomatic intracranial haemorrhage and ischaemic stroke; as cerebral microbleed burden increases, the relative risk (aHR) of intracranial haemorrhage rises more steeply than that of ischaemic stroke. Our most important new finding is that, regardless of cerebral microbleed burden and distribution (ie, mixed, deep, or lobar), or the type of antithrombotic treatment received (oral anticoagulants or antiplatelet therapy), the absolute risk of ischaemic stroke is consistently substantially higher than that of intracranial haemorrhage.

As well as confirming the association between cerebral microbleeds and both recurrent ischaemic stroke and symptomatic intracranial haemorrhage found in smaller cohorts of patients with ischaemic stroke and transient ischaemic attack treated with antiplatelet drugs^28^ or oral anticoagulants,^27^, ^57^, ^35^ the large number of participants has improved the precision of our estimates of stroke recurrence rates and relative hazards, while the inclusion of individual patient data allowed adjustment for potential confounding factors. Our study also adds new data for the important subgroups of patients with many (eg, ≥20) cerebral microbleeds, which cause the most clinical concern and could not be addressed by any of the previously published meta-analyses. The association of cerebral microbleeds with a consistently higher rate of ischaemic stroke than intracranial haemorrhage suggests that cerebral microbleeds are a marker for cerebral small vessel diseases that can cause not only intracranial haemorrhage, but also ischaemic stroke. Although it has been inferred that cerebral microbleeds are a marker of direct extravasation of red blood cells from arterioles and capillaries damaged by bleeding-prone arteriopathies, alternative non-haemorrhagic mechanisms include ischaemia-mediated iron store release by oligodendrocytes^10^ or phagocytosis of red cell microemboli into the perivascular space.^11^ A report of haemorrhagic transformation of small acute microinfarcts into cerebral microbleeds provides direct evidence that cerebral microbleeds can result from ischaemic mechanisms.^13^ These varied mechanisms underlying cerebral microbleeds might explain why even patients at the highest risk of intracranial haemorrhage still have a higher absolute risk of ischaemic stroke. Moreover, patients with cerebral microbleeds often have multiple vascular risk factors, so are at risk of not only small vessel ischaemic stroke but also other ischaemic stroke subtypes.^58^ Patients with cerebral microbleeds usually also have white matter hyperintensities, which are associated with the risk of recurrent stroke, death, and poor functional outcome after ischaemic stroke^59^ and might also contribute to the increased risk of ischaemic stroke associated with cerebral microbleeds.

We found no evidence that a strictly lobar pattern of cerebral microbleeds (fulfilling the Boston criteria for probable CAA,^5^ causing clinical concern for intracranial bleeding risk^35^) is associated with the risk of intracranial haemorrhage or ischaemic stroke. These findings might reflect low diagnostic accuracy when using cerebral microbleeds for diagnosis of CAA in patients without intracerebral haemorrhage or dementia,^60^ rather than a true absence of any association of CAA with intracranial haemorrhage. Furthermore, the aHRs for intracranial haemorrhage associated with lobar cerebral microbleeds (compared with patients without lobar cerebral microbleeds [including none]) were closer to those associated with deep or mixed cerebral microbleeds (compared with patients without deep or mixed cerebral microbleeds [including none]).

Our results differ from some previous observations in smaller cohorts. First, in contrast to a smaller two-centre study,^29^ we did not find that the risk of intracranial haemorrhage approached the risk of ischaemic stroke after 1 year. Rather, we found that the rate of ischaemic stroke was consistently higher than that of intracranial haemorrhage, and the aHRs associated with cerebral microbleeds for both ischaemic stroke and intracranial haemorrhage remained stable over time. Second, our data indicate a smaller increase in the relative risk of intracranial haemorrhage for patients with five or more cerebral microbleeds than reported in a previous smaller meta-analysis,^28^ but our much larger individual participant sample size allowed us to investigate high cerebral microbleed burdens (five or more, ten or more, and 20 or more) with adjustment for confounders and greater statistical precision and power.

The comparatively low frequency of symptomatic intracranial haemorrhage after ischaemic stroke or transient ischaemic attack and the consistently higher risk of recurrent ischaemic stroke make randomised controlled trials of antithrombotic treatment (themselves proven in large randomised trials) guided by cerebral microbleeds challenging. However, ongoing and future randomised controlled trials should provide further insights. The MRI substudy in the RESTART trial^61^ of antiplatelet therapy after intracerebral haemorrhage excluded all but a very modest harmful effect of antiplatelet therapy on recurrent intracerebral haemorrhage in the presence of cerebral microbleeds, but also illustrates how very large sample sizes are probably required to identify statistically significant interactions in smaller cerebral microbleed subgroups in current (eg, the MRI substudy of NAVIGATE ESUS [NCT02313909]) and future randomised controlled trials. Nevertheless, our large collaborative pooled analysis provides the best available evidence on the associations of cerebral microbleeds with subsequent intracranial haemorrhage and ischaemic stroke after ischaemic stroke or transient ischaemic attack.

We included data from a worldwide collaborative network, making our results globally generalisable. The large individual patient dataset provides high statistical power and precision for risk estimates, allowing us to explore associations with several clinically important primary outcomes, while adjusting for important prognostic variables to minimise confounding. Included cohorts used validated rating instruments for cerebral microbleeds, and we adjusted for the use of different MRI sequences (T2* GRE or SWI) to detect cerebral microbleeds, which accounts for the higher sensitivity of SWI for detecting cerebral microbleeds compared with T2* GRE.^62^ We followed a published statistical analysis plan and confirmed our findings in a two-stage meta-analysis, indicating the robustness of our results.

In terms of limitations, our observational design has potential for selection bias and confounding of antithrombotic therapy by indication or unmeasured physician factors; thus, the relative hazards (aHRs) for intracranial haemorrhage and ischaemic stroke must be interpreted with caution. To definitively establish whether cerebral microbleeds modify the net clinical benefit of antithrombotic drugs would require a randomised controlled trial. Many of the included studies did not formally adjudicate events. The requirement for MRI-suitable patients probably led to the inclusion of less severe strokes than an unselected population. Even with the many individual patients included, we could not precisely estimate risks associated with an extremely large number of cerebral microbleeds (eg, ≥50), but such patients are very rare in clinical practice. Although we adjusted for known prognostic variables, residual confounding secondary to unknown or uncontrolled factors such as stroke mechanism could still have affected our results. Furthermore, we were unable to include some candidate variables in our multivariable models because they were not sufficiently widely available across all participating cohorts (eg, white matter hyperintensities, MRI field strength, diabetes, ischaemic heart disease, renal function, and statin use on discharge). Our analyses did not formally assess net clinical benefit, accounting for the greater severity of intracranial haemorrhage compared with recurrent ischaemic stroke.

In summary, our large-scale pooled analysis in patients with recent ischaemic stroke or transient ischaemic attack found that the absolute risk of ischaemic stroke is consistently higher than that of intracranial haemorrhage, regardless of the number or anatomical distribution of cerebral microbleeds. However, cerebral microbleeds are associated with a greater relative hazard (aHR) for intracranial haemorrhage than ischaemic stroke; further studies are needed to establish the usefulness of neuroimaging biomarkers, including cerebral microbleeds, in improving risk prediction scores for intracranial haemorrhage and ischaemic stroke.

**This online publication has been corrected. The corrected version first appeared at thelancet.com/neurology on July 12, 2019, with subsequent corrections on January 3, 2020**
