# Correction to *Lancet Neurol* 2019; 18: 653–65

**DOI:** 10.1016/S1474-4422(19)30276-5

**Published:** 2019-09

*Wilson D, Ambler G, Lee K-J, et al. Cerebral microbleeds and stroke risk after ischaemic stroke or transient ischaemic attack: a pooled analysis of individual patient data from cohort studies.* Lancet Neurol *2019;* **18:** *653–65*—In this Article, the upper bound of the 95% CI for the adjusted hazard ratio for ischaemic stroke for patients with 20 or more cerebral microbleeds has been corrected to 2·82 in the summary and table 2. This correction has been made to the online version as of July 12, 2019.

