# Supplementary material for: Cerebral microbleeds and stroke risk after ischaemic stroke or transient ischaemic attack: a pooled analysis of individual patient data from cohort studies
Source: Lancet Neurol. 2019 Jul;18(7):653–65. doi: 10.1016/S1474-4422(19)30197-8 (PMC6562236; doi:10.1016/S1474-4422(19)30197-8)
Supplement: Supplementary appendix [file mmc1.pdf]

THE LANCET
Neurology

Supplementary appendix

This appendix formed part of the original submission and has been peer reviewed. We post it as supplied by the authors.

Supplement to: Wilson D, Ambler G, Lee K-J, et al. Cerebral microbleeds and stroke risk after ischaemic stroke or transient ischaemic attack: a pooled analysis of individual patient data from cohort studies. *Lancet Neurol* 2019; published online May 23. http://dx.doi.org/10.1016/S1474-4422(19)30197-8.

Cerebral microbleeds and stroke risk after ischaemic stroke or transient ischaemic attack: a pooled analysis of individual patient data from cohort studies

Online appendix

Table of Contents

Page 3	List of collaborators
Page 4	Table 1: Risk of bias table for the included cohorts
Page 6	Table 2: Baseline characteristics of patients with and without cerebral microbleeds (CMBs)
Page 7	Table 3: Baseline characteristics of patients with and without any composite event (intracranial haemorrhage or ischaemic stroke) during follow-up
Page 8	Table 4: Baseline characteristics of patients with and without intracranial haemorrhage during follow-up
Page 9	Table 5: Baseline characteristics of patients with and without ischaemic stroke during follow-up
Page 10	Figure 1: Kaplan Meier curves for CMB distributions and outcomes: A) Composite outcome; B) Intracranial haemorrhage; C) Ischaemic stroke
Page 13	Figure 2: Kaplan Meier curves for CMB presence and burden in (A) Patients treated with oral anticoagulants (with or without antiplatelet drugs) (n=7,737); and (B) Patients treated with antiplatelet drugs only (n=11,520)
Page 15	Figure 3: Forest plots for each outcome for all cohorts
Page 16	Table 6: Rates and adjusted Hazard Ratios (aHRs) for outcome events according to baseline CMBs in patients treated with antiplatelets only
Page 17	Secondary outcomes and sensitivity analyses
Page 19	Table 7: Baseline characteristics of patients according to whether they received treatment with antithrombotic drugs (antiplatelet drugs or oral anticoagulants)

List of collaborators

CROMIS-2: Kirsty Harkness, Louise Shaw, Jane Sword, Azlisham Mohd Nor, Pankaj Sharma, Deborah Kelly, Frances Harrington, Marc Randall, Matthew Smith, Karim Mahawish, Abduelbaset Elmarim, Bernard Esisi, Claire Cullen, Arumug Nallasivam, Christopher Price, Adrian Barry, Christine Roffe, John Coyle, Ahamad Hassan, Jonathan Birns, David Cohen, L Sekaran, Adrian Parry-Jones, Anthea Parry, David Hargroves, Harald Proschel, Prabel Datta, Khaled Darawil, Aravindakshan Manoj, Mathew Burn, Chris Patterson, Elio Giallombardo, Nigel Smyth, Syed Mansoor, Ijaz Anwar, Rachel Marsh, Sissi Ispoglou, Dinesh Chadha, Mathuri Prabhakaran, Sanjeevikumar Meenakishundaram, Janice O'Connell, Jon Scott, Vinodh Krishnamurthy, Prasanna Aghoram, Michael McCormick, Nikola Sprigg, Paul O'Mahony, Martin Cooper, Lillian Choy, Peter Wilkinson, Simon Leach, Sarah Caine, Ilse Burger, Gunaratam Gunathilagan, Paul Guyler, Hedley Emsley, Michelle Davis, Dulka Manawadu, Kath Pasco, Maam Mamun, Robert Luder, Mahmud Sajid, Ijaz Anwar, James Okwera, Elizabeth Warburton, Kari Saastamoinen, Timothy England, Janet Putterill, Enrico Flossman, Michael Power, Krishna Dani, David Mangion, Appu Suman, John Corrigan, Enas Lawrence, Djamil Vahidassr, Clare Shakeshaft, Martin M Brown , Andreas Charidimou, Hannah Cohen, Gargi Banerjee, Henry Houlden, Mark J White, Tarek A Yousry, Kirsty Harkness, Enrico Flossmann, Nigel Smyth, Louise J Shaw, Elizabeth Warburton, Keith W Muir. **Bern:** Marwan El-Koussy, Pascal Gratz. **TABASCO:** Jeremy Molad, Amos D Korczyn, Efrat Kliper. **PERFORM- MRI:** Philippe Maeder, Achim Gass, Chahin Pachai, Luc Bracoub, Marie-Yvonne Douste-Blazy, Marie Dominique Fratacci, Eric Vicaut. **SAMURAI NVAF:** Shoichiro Sato, Kaori Miwa, Kyohei Fujita, Toshihiro Ide. **Monash Stroke:** Henry Ma, John Ly, Shaloo Singhal, Ronil Chandra, Lee-anne Slater, Cathy Soufan, Christopher Moran. **Basel TIA and NOACISP:** Christopher Traenka, Sebastian Thilemann, Joachim Fladt, Henrik Gensicke, Leo H Bonati. **SNUBH Stroke cohort:** Beom Joon Kim, Moon-Ku Han, Jihoon Kang, Eunbin Ko, Mi Hwa Yang, Myung Suk Jang. **BIOSTROKE/TIA-Dublin:** Sean Murphy, Fiona Carty, Layan Akijian, John Thornton, Mark Schembri. **CASPER:** Elles Douven. **HERO:** Raquel Delgado-Mederos, Rebeca Marín, Pol Camps-Renom, Daniel Guisado-Alonso, Fidel Nuñez, Santiago Medrano-Martorell, Elisa Merino. **HAGAKURE:** Kotaro Iida, Syuhei Ikeda, Masashi Nishihara, Hiroyuki Irie. **Orken**: Derya Selcuk Demirelli. **CATCH:** Jayesh Modi Medanta, Charlotte Zerna. **MSS2:** Maria C Valdés Hernández, Paul Armitage, Anna K Heye, Susana Muñoz-Maniega, Eleni Sakka, Michael J Thrippleton, Martin Dennis. **Sainte-Anne:** Ysoline Beigneux, Mauro Silva. **Singapore:** Narayanaswamy Venketasubramanian. **HKU:** Shu Leung Ho, Raymond Tak Fai Cheung, Koon Ho Chan, Kay Cheong Teo, Edward S Hui, Joseph Shiu Kwong Kwan, Richard S.K. Chang, Man Yu Tse, Chu Peng Hoi, Chung Yan Chan, Oi Ling Chan, Ryan Hoi Kit Cheung, Edmund Ka Ming Wong. **IPAAC:** Kam Tat Leung, Suk Fung Tsang, Hing Lung Ip, Sze Ho Ma, Karen Ma, Wing Chi Fong, Siu Hung Li, Richard Li, Ping Wing Ng, Kwok Kui Wong, Wenyan Liu, Lawrence Wong. **MICRO**: Lino Ramos, Els De Schryver, Joost Jöbsis, Jaap van der Sande, Paul Brouwers, Yvo Roos, Jan Stam, Stef Bakker, Henk Verbiest, Wouter Schoonewille, Cisca Linn, Leopold Hertzberger, Maarten van Gemert, Paul Berntsen. **PARISK**: Jeroen Hendrikse, Paul J. Nederkoorn, Werner Mess, Peter J. Koudstaal. **SIGNaL:** Alexander Leff, Nicholas Ward, Parashkev Nachev, Richard J Perry, Hatice Ozkan, John Mitchell.

Table 1: Risk of bias assessment for the included cohorts

Cohort	Selection of cohorts	Assessment of exposure	Outcomes of interest not present at study inception?	Adjusting for variables associated with outcome	Assessment of presence/ absence of prognostic variables	Confidence in assessment of outcome	Follow up adequate?	Co-interventions similar between the groups?
**CROMIS-2**	++	++	++	++	++	++	++	+
**HBS**	++	++	++	++	++	++	++	+
**Bern**	++	++	++	++	++	+	+	+
**CU-STRIDE**	++	++	++	++	++	++	++	+
**TABASCO**	++	++	++	++	++	++	++	+
**Graz**	++	+	++	++	++	+	+	+
**PERFORM-MRI**	++	+	++	++	++	++	++	++
**PARISK**	++	++	++	++	++	++	++	++
**SAMURAI**	++	++	++	++	++	++	++	+
**RUNDMC**	++	++	++	++	++	++	++	+
**Wuerzburg**	++	++	++	++	++	+	++	++
**Monash Stroke**	++	++	++	++	++	++	++	+
**Basal TIA**	++	++	++	++	++	++	++	++
**Yonsei**	++	++	++	++	++	+	++	++
**SNUBH Stroke Cohort**	++	++	++	++	++	+	++	+
**BIOSTROKE/TIA**	++	++	++	++	++	+	++	+
**Kushiro City**	++	++	++	++	++	++	++	++
**Soo**	++	++	++	++	++	++	++	+
**CASPER**	++	+	++	++	++	++	++	+
**HERO**	++	++	++	++	++	++	++	++
**HAGAKURE**	++	++	++	++	++	++	++	++
**Leuven**	++	++	++	++	++	++	++	+
**NOACISP**	++	+	++	++	++	++	++	++
**Min Lou**	++	++	++	++	++	++	+	+
**MICRO**	++	++	++	++	++	++	++	+
**Orken**	++	++	++	++	++	+	++	+
**CATCH**	++	++	++	++	++	++	++	+
**MSS2**	++	++	++	++	++	++	+	++
**Sainte-Anne**	++	++	++	++	++	++	++	+
**STROKEDEM**	++	++	++	++	++	++	++	++
**Singapore**	++	++	++	++	++	+	++	++
**Future Study**	++	+	++	++	++	++	++	+
**Heidelberg**	++	++	++	++	++	+	++	+
**NNI**	++	++	++	++	++	++	++	+
**OXVASC**	++	++	++	++	++	++	++	++
**HKU**	++	++	++	++	++	++	++	++
**IPAAC Warfarin**	++	++	++	++	++	++	++	++
**SIGNaL**	++	++	++	++	++	++	++	++

Four-point risk of bias scale: ++ Low risk of bias, + minor risk of bias, -some risk of bias, -- high risk of bias

Studies were not eligible for inclusion if any of the criteria below were scored as - or --

Key for risk of bias assessment criteria

Selection of cohorts: ++ if exposed and unexposed participants are recruited from the same populations over the same time periods

Assessment of exposure: ++ use of validated scale for rating CMBs; + use of defined criteria for CMBS

Outcome of interest not present at study inception? ++ for all studies (all outcomes of interest require re-hospitalisation for an outcome event)

Adjusting for variables associated with outcome: ++ or + for all studies as we defined prespecified criteria for study entry

Assessment of presence or absence of prognostic variables: ++ if studies used pre-defined criteria for hypertension, diabetes, etc.

Confidence in assessment of outcome: ++ if outcome adjudicated blinded to CMBs; + if outcome was not adjudicated but blinded to CMBs and with imaging confirmation

Follow up adequate? ++ if less than 5% loss to follow up; + if less than 20% loss to follow up. As we used survival analysis, censoring is unlikely to introduce bias for studies with short follow up times

Co-interventions similar between the groups? ++ if antithrombotic decisions were made blinded to CMB presence; + if antithrombotic decisions were not blinded to CMB presence, but antithrombotic choices were similar among the CMB and non-CMB groups

Table 2. Baseline characteristics of patients with and without cerebral microbleeds (CMBs)

Variable	CMBs present n=5649	CMBs absent n=14673	p value
Age, mean (SD)	73 (13)	69 (11)	<0·0001
Sex (female), n (%)	2314 (41)	6279/14665 (43)	0·0166
Hypertension, n (%)	4338/5639 (78)	9977/14632 (68)	<0·0001
Previous stroke n (%)	1217/5644 (22)	2082/14646 (14)	<0·0001
Diabetes, n (%)	1380/5247 (26)	3121/12921 (24)	0·0024
Regular anticoagulants prior to index stroke, n (%)	392/3832 (10)	833/10335 (8)	<0·0001
Regular antiplatelets prior to index stroke, n (%)	1427/3829 (37)	3340/10315 (32)	<0·0001
Ischaemic heart disease, n (%)	829/5322 (16)	1779/13520 (13)	<0·0001
Antiplatelets at discharge, n (%)	3749 (66)	9606/14665 (66)	0·239
Anticoagulants at discharge, n (%)	2062 (37)	5675 (39)	0·00381
Statins on discharge, n (%)	2859/4377 (65)	7044/11100 (63)	0·0300

Table 3. Baseline characteristics of patients with and without any composite event (intracranial haemorrhage or ischaemic stroke) during follow up

Variable	All patients (n=20332)	Any composite event n=1461	No composite event n=18861	p value
**Age, mean (SD)**	70 (12)	72 (12)	70 (13)	<0·0001
**Sex (female), n (%)**	8593/20314 (42)	621 (43)	7972/18853 (42)	0·870
**Hypertension, n (%)**	14365/20271 (71)	1102/1460 (75)	13263/18811 (71)	<0·0001
**Previous stroke, n (%)**	3299/20290 (16)	371/1459 (25)	2928/18831 (16)	<0·0001
**Diabetes, n (%)**	4501/18168 (25)	375/1301 (29)	4126/16867 (24)	<0·0001
**Regular anticoagulants prior to index stroke, n (%)**	1225/14167 (9)	116/1043 (11)	1109/13124 (8)	0·00313
**Regular antiplatelets prior to index stroke, n (%)**	4767/14144 (34)	401/1041 (39)	4366/13103 (33)	0·000635
**Ischaemic heart disease, n (%)**	2608/18842 (14)	248/1409 (18)	2360/17433 (14)	<0·0001
**Cerebral microbleed presence, n (%)**	5649 (28)	542 (37)	5107 (27)	<0·0001
**Antiplatelets at discharge, n (%)**	12715/20312 (62)	1016 (70)	12339/18852 (65)	0·002
**Anticoagulants at discharge, n (%)**	7690 (38)	500 (34)	7237/18858 (38)	0·002
**Statins on discharge, n (%)**	9903/15477 (64)	652/1079 (60)	9251/14398 (64)	0·0116

Table 4. Baseline characteristics of patients with and without intracranial haemorrhage during follow up

Variable	Intracranial haemorrhage present n=189	Intracranial haemorrhage absent n=16778	p value
Age, mean (SD)	75 (11)	71 (12)	<0·0001
Sex (female), n (%)	84 (44)	7162/16770 (43)	0·631
Hypertension, n (%)	146 (77)	11895/16728 (71)	0·0638
Previous stroke n (%)	52/188 (28)	2760/16747 (16)	<0·0001
Diabetes, n (%)	46/174 (26)	3437/14639 (23)	0·360
Regular anticoagulants prior to index stroke, n (%)	15/174 (9)	1210/13993 (9)	0·990
Regular antiplatelets prior to index stroke, n (%)	62/173 (36)	4705/13971 (34)	0·550
Ischaemic heart disease, n (%)	30/182 (16)	2294/15503 (15)	0·575
Cerebral microbleed presence, n (%)	98 (52)	4385 (26)	<0·0001
Antiplatelets at discharge, n (%)	108 (57)	10143/16769 (60)	0·350
Anticoagulants at discharge, n (%)	91 (48)	7021 (42)	0·081
Statins on discharge, n (%)	75/135 (56)	7106/11988 (59)	0·382

Table 5: Baseline characteristics of patients with and without ischaemic stroke during follow up

Variable	Ischaemic stroke present n=1113	Ischaemic stroke absent n=15854	p value
**Age, mean (SD)**	72 (11)	71 (12)	0·0004
**Sex (female), n (%)**	467 (42)	6779/15846 (43)	0·592
**Hypertension, n (%)**	836/1112 (75)	11205/15805 (71)	0·0230
**Previous stroke n (%)**	286/1112 (26)	2526/15823 (16)	<0·0001
**Diabetes, n (%)**	280/968 (29)	3203/13845 (23)	<0·0001
**Regular anticoagulants prior to index stroke, n (%)**	103/882 (12)	1122/13285 (8)	0·000941
**Regular antiplatelets prior to index stroke, n (%)**	347/881 (39)	4420/13263 (33)	0·000228
**Ischaemic heart disease, n (%)**	200/1068 (19)	2124/14419 (15)	0·000418
**Cerebral microbleed presence, n (%)**	380 (43)	4103 (26)	<0·0001
**Antiplatelets at discharge, n (%)**	673 (60)	8938/15844 (56)	0·00832
**Anticoagulants at discharge, n (%)**	376 (35)	6728 (42)	<0·0001
**Statins on discharge, n (%)**	434/781 (56)	6747/11342 (59)	0·0312

Figure 1: Kaplan Meier curves for CMB distributions and outcomes: A) Composite outcome; B) Intracranial haemorrhage; C) Ischaemic stroke

A

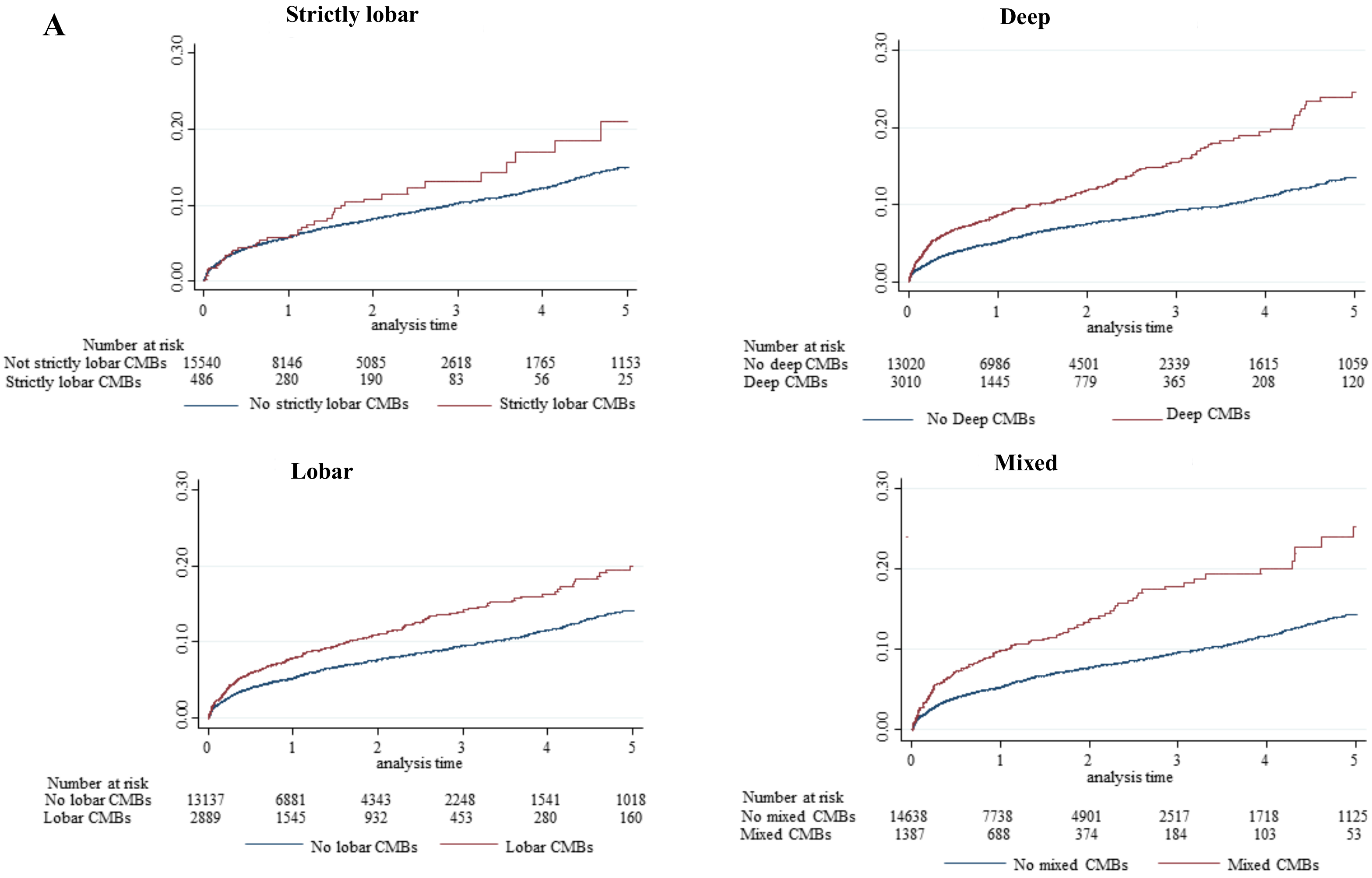

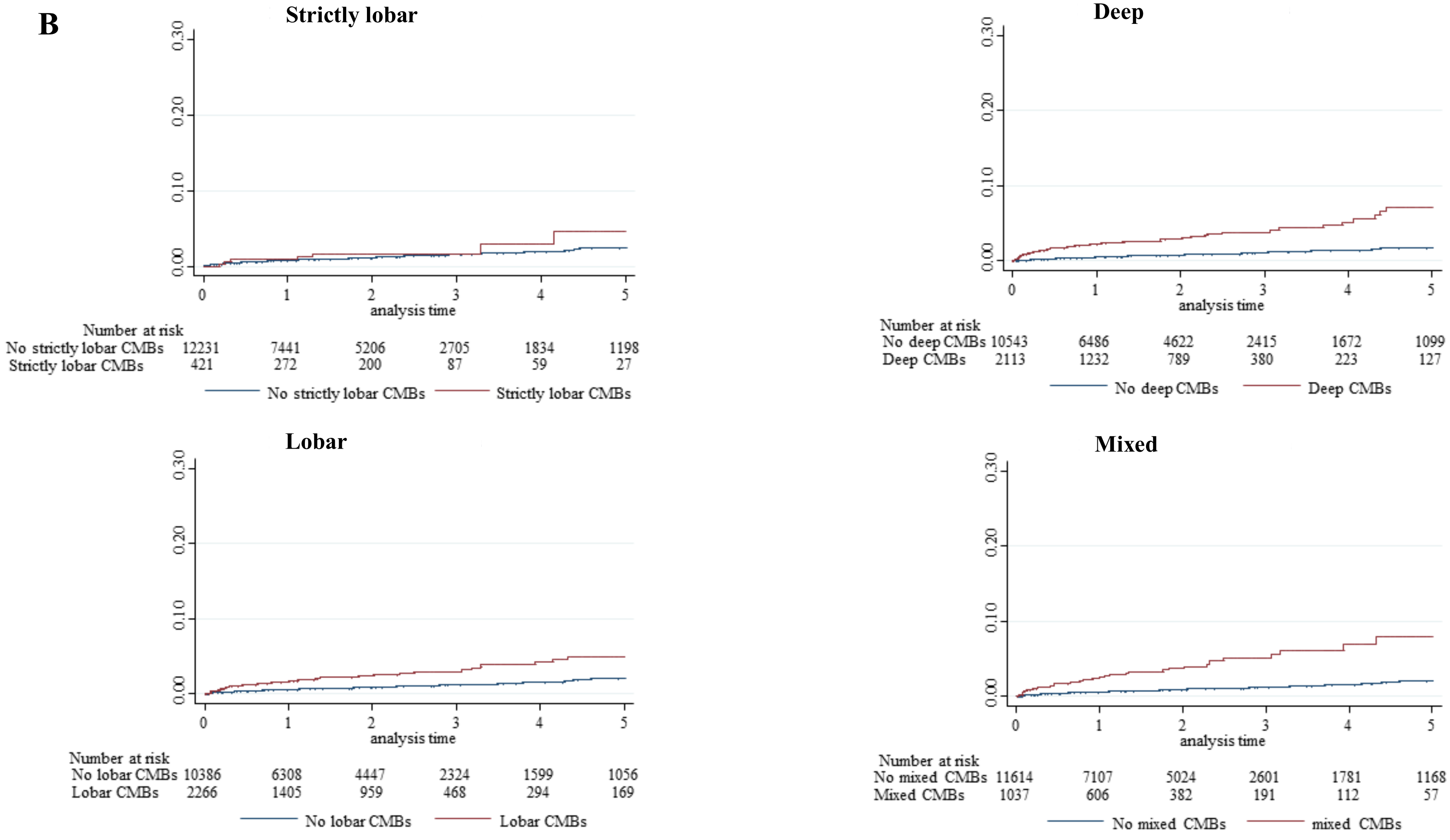
B
Strictly lobar
0.00
0.10
0.20
0.30
0 1 2 3 4 5
analysis time
Number at risk
No strictly lobar CMBs 12231 7441 5206 2705 1834 1198
Strictly lobar CMBs 421 272 200 87 59 27
No strictly lobar CMBs
Strictly lobar CMBs
Deep
0.00
0.10
0.20
0.30
0 1 2 3 4 5
analysis time
Number at risk
No deep CMBs 10543 6486 4622 2415 1672 1099
Deep CMBs 2113 1232 789 380 223 127
No deep CMBs
Deep CMBs
Lobar
0.00
0.10
0.20
0.30
0 1 2 3 4 5
analysis time
Number at risk
No lobar CMBs 10386 6308 4447 2324 1599 1056
Lobar CMBs 2266 1405 959 468 294 169
No lobar CMBs
Lobar CMBs
Mixed
0.00
0.10
0.20
0.30
0 1 2 3 4 5
analysis time
Number at risk
No mixed CMBs 11614 7107 5024 2601 1781 1168
Mixed CMBs 1037 606 382 191 112 57
No mixed CMBs
mixed CMBs

C

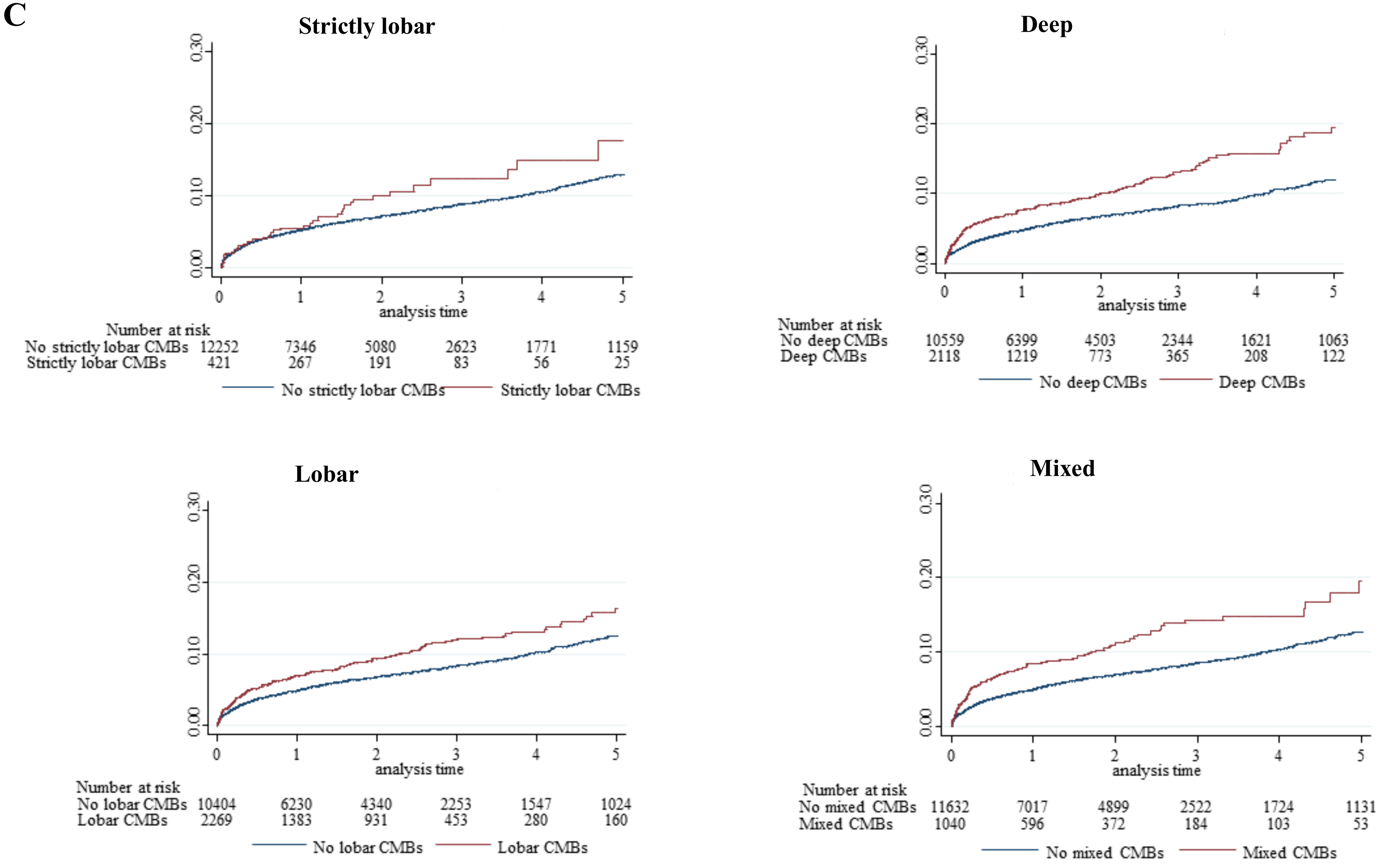
Strictly lobar
0.00
0.10
0.20
0.30
0
1
2
3
4
5
analysis time
Number at risk
No strictly lobar CMBs 12252 7346 5080 2623 1771 1159
Strictly lobar CMBs 421 267 191 83 56 25
No strictly lobar CMBs
Strictly lobar CMBs
Deep
analysis time
Number at risk
No deep CMBs 10559 6399 4503 2344 1621 1063
Deep CMBs 2118 1219 773 365 208 122
No deep CMBs
Deep CMBs
Lobar
analysis time
Number at risk
No lobar CMBs 10404 6230 4340 2253 1547 1024
Lobar CMBs 2269 1383 931 453 280 160
No lobar CMBs
Lobar CMBs
Mixed
analysis time
Number at risk
No mixed CMBs 11632 7017 4899 2522 1724 1131
Mixed CMBs 1040 596 372 184 103 53
No mixed CMBs
Mixed CMBs

Figure 2: Kaplan Meier curves for CMB presence and burden in (A) Patients treated with oral anticoagulants (with or without antiplatelet drugs) (n=7,737); and (B) Patients treated with antiplatelet drugs only (n=11,520)

A

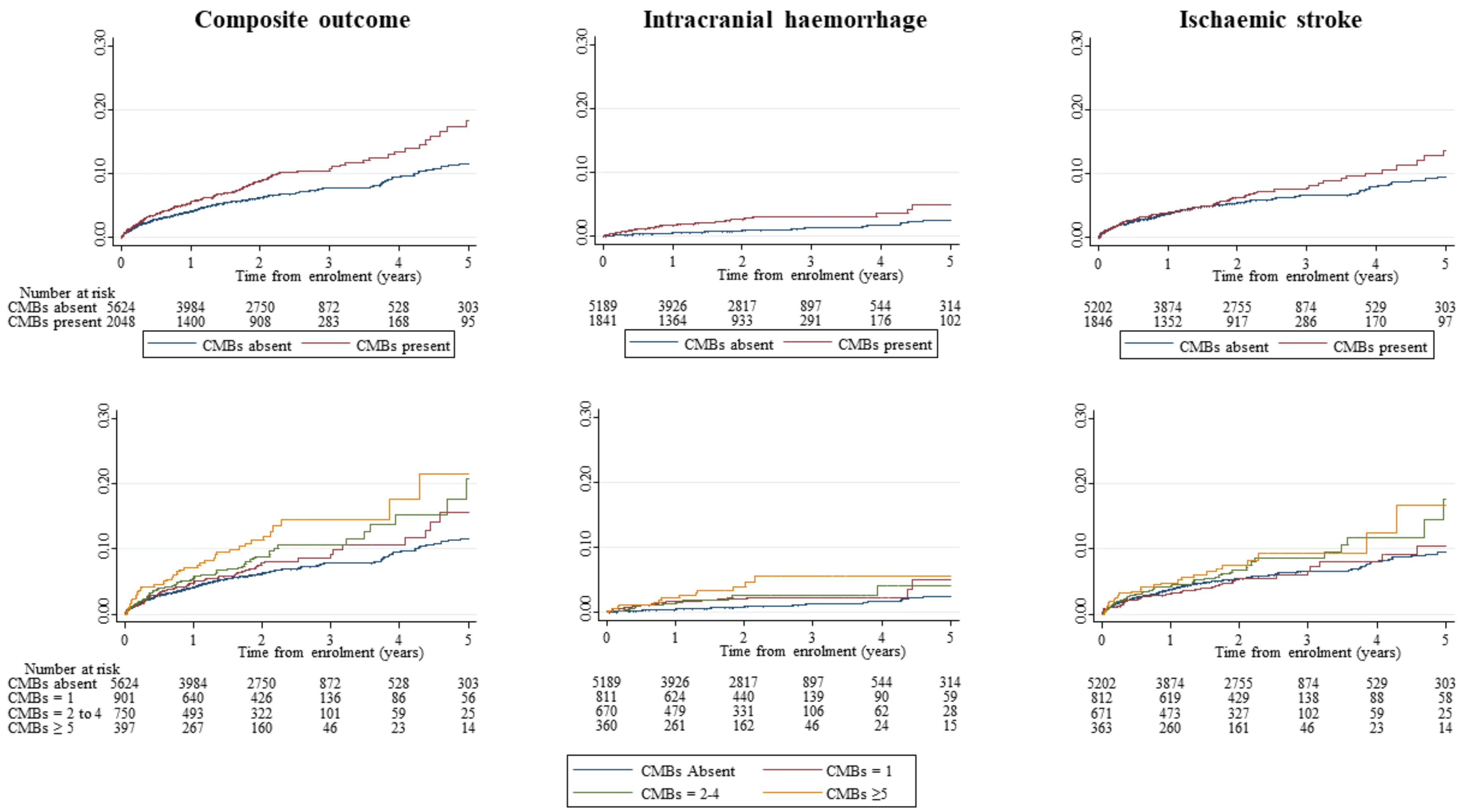

B

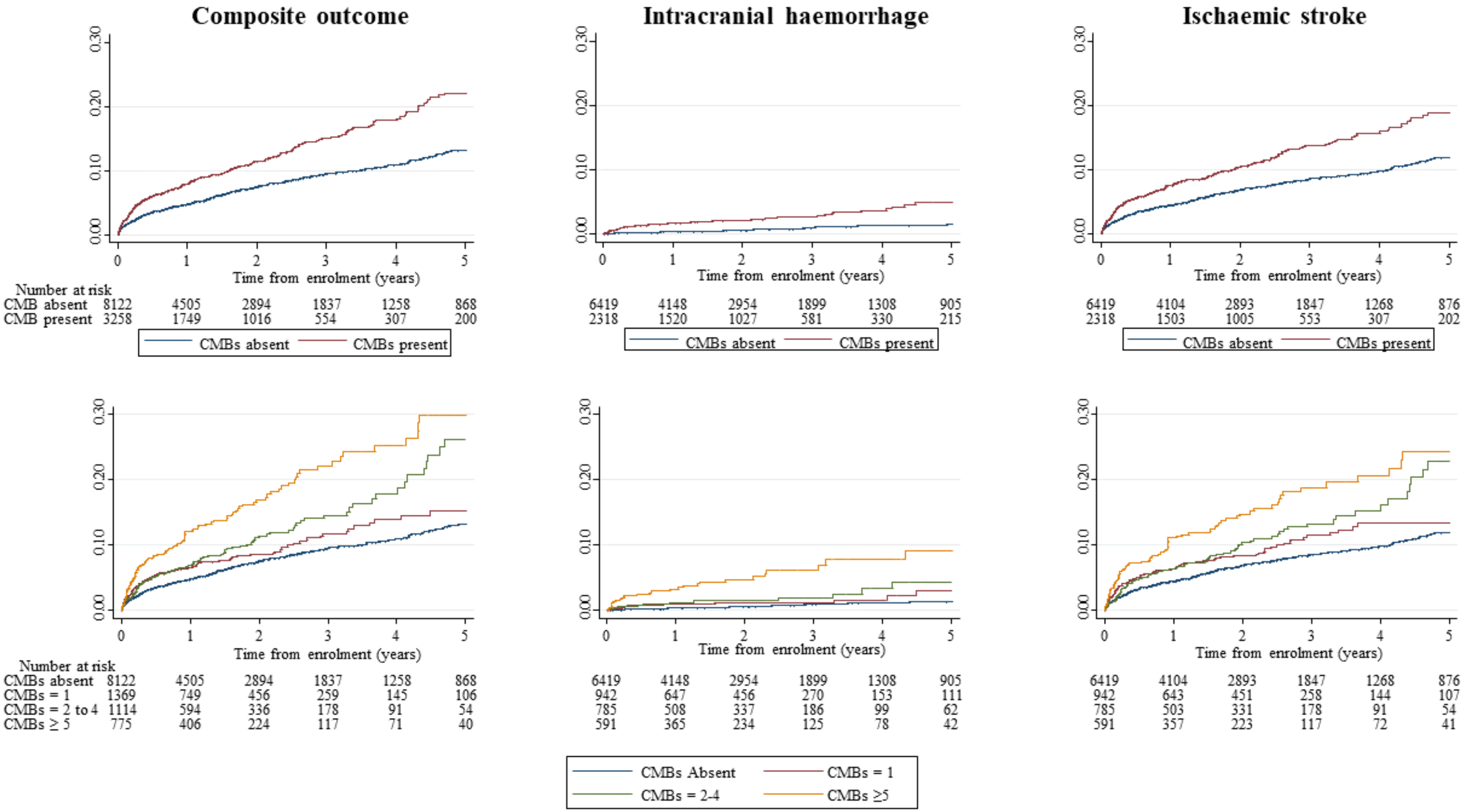
Composite outcome
Intracranial haemorrhage
Ischaemic stroke
0.00
0.10
0.20
0.30
0 1 2 3 4 5
Time from enrolment (years)
Number at risk
CMB absent 8122 4505 2894 1837 1258 868
CMB present 3258 1749 1016 554 307 200
6419 4148 2954 1899 1308 905
2318 1520 1027 581 330 215
6419 4104 2893 1847 1268 876
2318 1503 1005 553 307 202
CMBs absent
CMBs present
Number at risk
CMBs absent 8122 4505 2894 1837 1258 868
CMBs = 1 1369 749 456 259 145 106
CMBs = 2 to 4 1114 594 336 178 91 54
CMBs ≥ 5 775 406 224 117 71 40
6419 4148 2954 1899 1308 905
942 647 456 270 153 111
785 508 337 186 99 62
591 365 234 125 78 42
6419 4104 2893 1847 1268 876
942 643 451 258 144 107
785 503 331 178 91 54
591 357 223 117 72 41
CMBs Absent
CMBs = 1
CMBs = 2-4
CMBs ≥5

Figure 3. Forest plots for each outcome for all cohorts

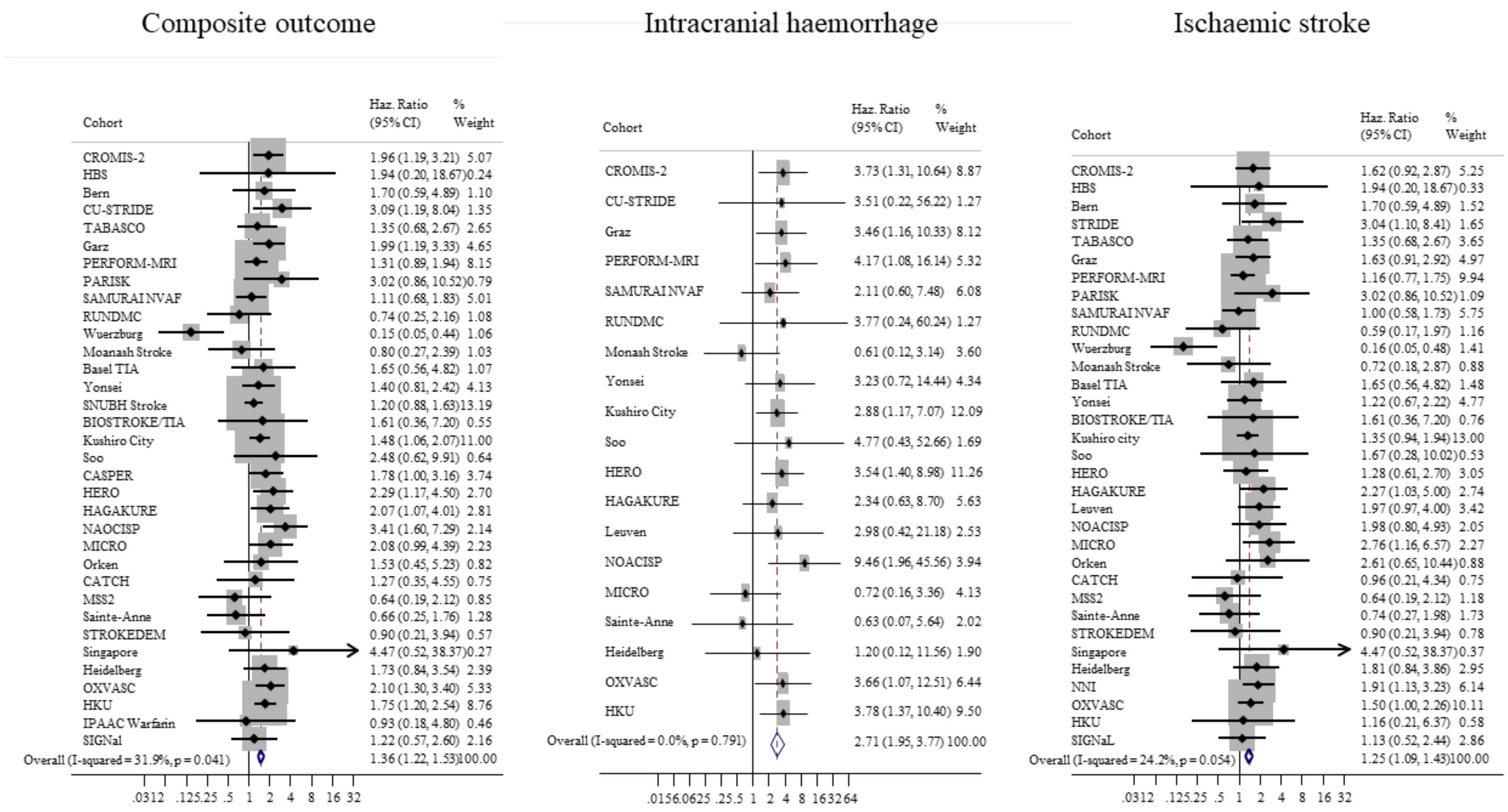

Table 6. Incidence and risk of outcome events according to baseline CMB in patients treated with antiplatelets only.

PATIENTS TREATED WITH ANTIPLATELET DRUGS ONLY (n=11520)												
	Composite of intracranial haemorrhage and ischaemic stroke (n=11312 in multivariable models)				Symptomatic intracranial haemorrhage (n=8670 in multivariable models)				Symptomatic ischaemic stroke (n=8670 in multivariable models)			
	n/time at risk (years)	Rate/1000 patient years (95% CI)	Absolute rate increase/ 1000 patient years (95% CI)	Adjusted Hazard ratio (95% CI)	n/time at risk (years)	Rate/1000 patient years (95% CI)	Absolute rate increase/ 1000 patient years (95% CI)	Adjusted Hazard ratio (95% CI)	n/time at risk (years)	Rate/1000 patient years (95% CI)	Absolute rate increase/ 1000 patient years (95% CI)	Adjusted Hazard ratio (95% CI)
**No CMB**	544/14798	37 (34 to 40)	*Reference*	*Reference*	41/13518	3 (2 to 4)	*Reference*	*Reference*	424/13290	32 (29 to 35)	*Reference*	*Reference*
**CMB present**	343/5301	65 (58 to 72)	28 (24 to 32)	1·38 (1·19 to 1·59)	52/4541	11 (9 to 15)	8 (7 to 11)	2·59 (1·68 to 4·00)	240/4442	54 (47 to 61)	22 (18 to 26)	1·32 (1·11 to 1·56)
**1 CMB**	114/2340	49 (40 to 59)	12 (6 to 19)	1·20 (0.97 to 1·47)	12/1984	6 (3 to 11)	3 (1 to 7)	1·60 (0·83 to 3·08)	82/1951	42 (33 to 52)	10 (4 to 17)	1·22 (0·96 to 1·55)
**2-4 CMBs**	111/1759	63 (52 to 76)	26 (18 to 36)	1·25 (1·02 to 1·55)	13/1487	9 (5 to 15)	6 (3 to 11)	1·82 (0·95 to 3·47)	78/1457	54 (42 to 67)	22 (13 to 32)	1·20 (0·94 to 1·55)
**≥5 CMBs***	118/1203	98 (81 to 117)	61 (47 to 77)	1·85 (1·50 to 2·28)	27/1069	25 (17 to 37)	22 (15 to 33)	5·69 (3·36 to 9·65)	80/1034	77 (61 to 96)	45 (32 to 61)	1·63 (1·27 to 2·09)
**≥10 CMBs***	59/593	99 (76 to 128)	62 (42 to 88)	1·82 (1·38 to 2·40)	16/519	31 (18 to 50)	28 (16 to 46)	6·81 (3·67 to 12·63)	37/511	72 (51 to 100)	40 (22 to 65)	1·47 (1·04 to 2·07)
**≥20 CMBs***	30/221	136 (92 to 194)	99 (58 to 154)	2·86 (1·97 to 4·96)	11/194	57 (28 to 101)	54 (26 to 97)	15·71 (7·69 to 32·11)	16/192	83 (48 to 135)	51 (19 to 100)	2·00 (1·20 to 3·32)
**Mixed CMBs**	102/1121	91 (74 to 110)	54 (40 to 70)	1·21 (0·96 to 1·52)	24/896	27 (17 to 40)	24 (15 to 36)	2·90 (1·64 to 5·13)	60/867	69 (53 to 89)	37 (21 to 54)	1·03 (0·76 to 1·39)
**Deep CMBs**	193/2320	83 (72 to 96)	46 (38 to 56)	1·22 (1·02 to 1·47)	34/1692	20 (14 to 28)	17 (12 to 24)	2·63 (1·54 to 4·47)	116/1647	70 (58 to 84)	38 (29 to 49)	1·11 (0·88 to 1·40)
**Lobar CMBs**	180/2423	74 (64 to 86)	37 (30 to 46)	1·26 (1·05 to 1·52)	31/2029	15 (10 to 22)	12 (8 to 18)	2·44 (1·44 to 4.15)	123/1973	62 (52 to 74)	30 (23 to 39)	1·26 (1·01 to 1·57)
**Probable CAA**	32/372	86 (59 to 121)	49 (25 to 81)	1·52 (1·07 to 2·18)	3/340	9 (2 to 26)	6 (0 to 22)	1·43 (0·44 to 4·59)	28/326	86 (57 to 124)	54 (28 to 89)	1·79 (1·21 to 2·63)

Footnote: CMB location HRs are compared to patients without CMBs in each location and are adjusted for CMB number in addition to our pre-specified variables, *overlapping categories

Secondary outcomes and sensitivity analyses

Secondary outcomes: death and vascular death

There were 2418 deaths, 484 of which were vascular. In multivariable analyses, CMB presence was not associated with all-cause death (aHR 1·03, 95% CI 0·94-1·12) or vascular death (aHR 0·97, 95% CI 0·79-1·19).

Ethnicity

There was no interaction between CMB and ethnicity (n=15123; 6743 white, 8380 Asian) for the risks of: the composite outcome of intracranial haemorrhage or ischaemic stroke (p-interaction=0·707); intracranial haemorrhage (p-interaction=0·537); or ischaemic stroke (p-interaction=0·654).

Age

There was no interaction between CMB and older age (4376 patients aged >80 years) for the risk of the composite outcome of intracranial haemorrhage or ischaemic stroke (p-interaction=0·538); intracranial haemorrhage (p-interaction=0·219); or ischaemic stroke (p-interaction=0·286).

Sensitivity analysis to quantify heterogeneity

Using a two-stage meta-analysis, the estimated risks associated with CMB presence were consistent with our main model for: the composite of intracranial haemorrhage or ischaemic stroke (heterogeneity (I^2=31.7%); intracranial haemorrhage ($I^{2=}$0%); and ischaemic stroke (I^2=24.2%). The forest plots are shown above, in appendix Figure 3.

Sensitivity analysis including white matter hyperintensities

23 cohorts, including 10,235 patients, provided ratings for WMH, which were moderate to severe (Fazekas grade≥2) in 3,105 (30%). Including WMH in multivariable models did not substantially change the aHR

associated with the presence of CMBs for the composite outcome (aHR 1.30, 95% CI 1.12-1.52), intracranial haemorrhage (aHR 2.44, 95% CI 1.68-3.53) or for ischaemic stroke (aHR 1.16, 95% CI 0.98-1.37).

Sensitivity analysis including only intracerebral, convexity subarachnoid and subdural intracranial haemorrhage

183 patients had a symptomatic intracranial haemorrhage during 32,847 patient-years of follow up. The risk of symptomatic intracranial haemorrhage was 2·59 (95% CI 1·91-3·50) times higher for patients with CMBs than patients without CMBs and rose with increasing CMB burden. Compared to no CMBs, aHRs were: 1·92 (95% CI 1·25-2·94) for 1 CMB; 2.02 (95% CI 1·30-3·16) for 2-4 CMBs; 4·88 (95% CI 3·29-7·25) for ≥5 CMBs; 5·87 (95% CI 3·56-9·66) for ≥ 10 CMBs; and 9·32 (95% CI 5·06-17·16) for ≥20 CMBs. These results are consistent with our primary findings.

Incidence of outcome events beyond the first year and change in risks over time

There were 102 intracranial haemorrhage events over 12,794 patient-years of follow up within the first year, and 87 symptomatic intracranial haemorrhages over 31,059 patient-years of follow-up after the first year. In patients with CMBs, the incidence of intracranial haemorrhage (per 1000 patients-years) was 18 (95% CI 14-23) within the first year, and 5 (95% CI 3-6) after the first year.

There were 696 ischaemic strokes over 12,873 patient -years of follow up within the first year, and 417 symptomatic ischaemic strokes during 30447 patient-years of follow up after the first year. In patients with CMBs the incidence of symptomatic ischaemic stroke within the first year was 70 (95% CI 62-80), and 18 (95% CI 15-21) after the first year. Accounting for death as a competing risk, we found no evidence for a change in risk over time associated with CMB presence for intracranial haemorrhage (subdistribution Hazard Ratio [SHR] 4·96, 95% CI 3·18-7·74 at day 0 vs. 4·81, 95% CI 3·15-7·35 after 1 year) or ischaemic stroke. (SHR 1·46, 95% CI 1·23-1·73 at day 0 vs. 1·49, 95% CI 1·27-1·75 after 1 year).

Table 7. Baseline characteristics of patients according to whether they received treatment with antithrombotic drugs (antiplatelet drugs or oral anticoagulants) following ischaemic stroke or TIA.

Variable	Antithrombotic drug treatment received n=1065	Antithrombotic drug treatment not received n=19257	p value
**Age mean (SD)**	72 (14)	70 (13)	<0·0001
**Sex (female), n (%)**	489 (46)	8104/19257 (42)	0·0074
**Hypertension, n (%)**	727/1051 (69)	13638/19220 (71)	0·215
**Previous ischaemic stroke, n (%)**	171/1044 (16)	2762/18156 (15)	0.101
**Previous intracranial haemorrhage, n (%)**	45/742 (6%)	319/16480 (2)	<0.0001
**Diabetes, n (%)**	261/905 (27)	4240/17203 (25)	0.0930
**Regular anticoagulants prior to index stroke, n (%)**	105/829 (13)	1120/13338 (8)	<0.0001
**Regular antiplatelets prior to index stroke, n (%)**	221/826 (27)	4546/13318 (34)	<0.0001
**Ischaemic heart disease, n (%)**	158/984 (16)	2450/17858 (14)	0·0387
**Stroke (vs. TIA) at study entry, n (%)**	967/1064 (91)	15901/19247 (83)	<0.0001
**Cerebral microbleed presence, n (%)**	306 (29)	5243 (28)	0.484
